# Supplementary material for: Reactant-dependent volcano trends in Pt-catalyzed cycloalkane dehydrogenation: orbital hybridization-guided design of active sites
Source: Nat Commun. 2025 Dec 9;17:90. doi: 10.1038/s41467-025-66782-w (PMC12770571; doi:10.1038/s41467-025-66782-w)
Supplement: Supplementary file 1 — Supplementary Information [file 41467_2025_66782_MOESM1_ESM.pdf]

Supporting Information

Reactant-Dependent Volcano Trends in Pt-Catalyzed Cycloalkane Dehydrogenation: Orbital Hybridization-Guided Design of Active Sites

Yongxiao Tuo [1,+], Jingying Qu [2,+], Huailu Sun [2], Qing Lu [2], Bin Wang [3,4,*], Hongwei Gai [1], Defu Yin [2], Xiang Feng [2,*] , De Chen [1,5,*]

[1] *State Key Laboratory of Heavy Oil Processing, College of New Energy, China University of Petroleum (East China), 66 West Changjiang Road, Qingdao, Shandong 266580, China*

[2] *College of Chemistry and Chemical Engineering, China University of Petroleum (East China), 66 West Changjiang Road, Qingdao, Shandong 266580, China*

[3] *Department of Chemical Engineering, Shaanxi Key Laboratory of Energy Chemical Process Intensification, Engineering Research Center of New Energy System Engineering and Equipment, Xi'an Jiaotong University, Xi'an, China*

[4] *Shaanxi HydroTransformer Energy Technologies Co., Ltd, Xi'an, China*

[5] *Department of Chemical Engineering, Norwegian University of Science and Technology, N-7491 Trondheim, Norway*

∗ Corresponding authors: dr. binwang@xjtu.edu.cn (Bin Wang), xiangfeng@upc.edu.cn (Xiang Feng), de.chen@ntnu.no (De Chen).

[+] These authors contributed equally to this work.

I. Supplementary Figures **6**
Figure S1. Textural structure characterization of the catalysts. **6**
Figure S2. XRD characterization of the catalysts. **7**
Figure S3. Schematic representation of Pt species distribution. **8**
Figure S4. Catalytic performance of Pt/MAO catalysts toward the dehydrogenation of various cycloalkanes. **9**
Figure S5. Temperature-dependent hydrogen release behavior of Pt/MAO catalysts toward the dehydrogenation of various cycloalkanes. **10**
Figure S6. Atomic-scale morphology of Pt nanoparticles supported on MAO. **11**
Figure S7. Correlation between Pt nanoparticle morphology and intrinsic catalytic activity in cycloalkane dehydrogenation. **12**
Figure S8. Residuals of least-squares fitting for Pt species quantification. **13**
Figure S9. Statistical confidence analysis of fitted Pt species fractions. **14**
Figure S10. Site-specific TOFs of Pt/MAO catalysts. **15**
Figure S11. EXAFS fitting of Pt L_{III}-edge spectra in k-space. **16**
Figure S12. EXAFS fitting of Pt L_{III}-edge spectra in R-space. **17**
Figure S13. Site-specific TOF of Pt/MAO catalysts estimated using the cuboctahedron model **18**
Figure S14. HAADF-STEM images and particle size distributions of Pt/Al_2O_3 catalysts. **19**
Figure S15. Hydrogen evolution activities of Pt/Al_2O_3 catalysts. **20**
Figure S16. Long-term stability of Pt/MAO catalysts. **21**
Figure S17. In-situ FTIR and CO-DRIFTS characterization of 0.05Pt/MAO catalyst. **22**
Figure S18. Structural characterization of spent 0.15Pt/MAO catalyst. **23**
Figure S19. Coke resistance characterization of Pt catalysts. **24**
Figure S20. Coke characterization of Pt/MAO catalyst. **25**
Figure S21. Arrhenius plots of Pt/MAO catalysts. **26**

Figure S22. Eyring plots of Pt/MAO catalysts. 27
Figure S23. Relationship between Pt coordination and apparent activation energy. ... 28
Figure S24. Entropic and enthalpic trends in dehydrogenation on Pt/MAO catalysts. 29
Figure S25. Kinetic dependence of reaction rates on partial pressures for cyclohexane dehydrogenation. 30
Figure S26. Kinetic dependence of reaction rates on partial pressures for methylcyclohexane dehydrogenation. 31
Figure S27. Kinetic dependence of reaction rates on partial pressures for decalin dehydrogenation. 32
Figure S28. Product inhibition effects in dehydrogenation. 33
Figure S29. Structural models of Pt species on the MAO support. 34
Figure S30. DFT-optimized geometries for cyclohexane dehydrogenation on Pt_1/MAO. 35
Figure S31. DFT-optimized geometries for methylcyclohexane dehydrogenation on Pt_1/MAO.. 36
Figure S32. DFT-optimized geometries for decalin dehydrogenation on Pt_1/MAO.... 37
Figure S33. DFT-optimized geometries for cyclohexane dehydrogenation on Pt_4/MAO. 38
Figure S34. DFT-optimized geometries for methylcyclohexane dehydrogenation on Pt_4/MAO. 39
Figure S35. DFT-optimized geometries for decalin dehydrogenation on Pt_4/MAO.... 40
Figure S36. DFT-optimized geometries for cyclohexane dehydrogenation on Pt_{13}/MAO. 41
Figure S37. DFT-optimized geometries for methylcyclohexane dehydrogenation on Pt_{13}/MAO. 42
Figure S38. DFT-optimized geometries for decalin dehydrogenation on Pt_{13}/MAO. . 43
Figure S39. DFT-optimized geometries for cyclohexane dehydrogenation on Pt(111). 44

Figure S40. DFT-optimized geometries for methylcyclohexane dehydrogenation on Pt(111). **45**

Figure S41. DFT-optimized geometries for decalin dehydrogenation on Pt(111). **46**

Figure S42. Energy trends for dehydrogenation on Pt catalysts. **47**

Figure S43. Rate-determining transition state for C-H activation. **48**

Figure S44. C_7H_8 demethylation on Pt_{13}/MAO and Pt(111). **49**

Figure S45. Correlation between coordination number, d-band center, and Pt 5d electron vacancy. **50**

Figure S46. C-C bond cleavage on Pt catalysts. **51**

Figure S47. Selectivity trends on Pt catalysts. **52**

Figure S48. Molecular geometries of dehydrogenation products. **53**

Figure S49. LUMO energy comparison. **54**

Figure S50. Electronic properties of dehydrogenation products. **55**

Figure S51. Relationship between Pt d-band center and LUMO energy along dehydrogenation pathways. **56**

Figure S52. Bonding strength analysis of Pt-C interactions during key reaction steps.. **57**

Figure S53. LUMO energy comparison of benzyltoluene isomers. **58**

Figure S54. LUMO energy comparison of dibenzyltoluene isomers.. **59**

Figure S55. Adsorption behavior of benzyltoluene isomers on Pt surfaces.. **60**

Figure S56. Adsorption behavior of dibenzyltoluene isomers on Pt surfaces. **61**

Figure S57. Adsorption energy trends of dehydrogenation products. **62**

Figure S58. Reactant structure-dependent dehydrogenation activity of Pt/MAO catalysts. **63**

Figure S59. Electronic matching between Pt active sites and aromatic product orbitals. **64**

II. Supplementary Tables **65**

Table S1. BET surface area, pore volume, and average pore diameter of Pt/MAO

catalysts with varying Pt loadings. **65**

Table S2. Physic-chemical characteristics of the as-prepared Pt/MAO catalysts. **66**

Table S3. Comparative analysis of cyclohexane dehydrogenation performance across reported catalysts **67**

Table S4. Comparative analysis of methylcyclohexane dehydrogenation performance across reported catalysts. **68**

Table S5. Comparative analysis of decalin dehydrogenation performance across reported catalysts. **69**

Table S6. Least-squares fitting details for determining the fractions of Pt species in 0.05Pt/MAO and 0.15Pt/MAO catalysts based on kinetic data. **70**

Table S7. EXAFS fitting parameters at the Pt L_{III}-edge for various Pt samples (S_0^2=0.90). **71**

III. Supplementary References **72**

I. Supplementary Figures

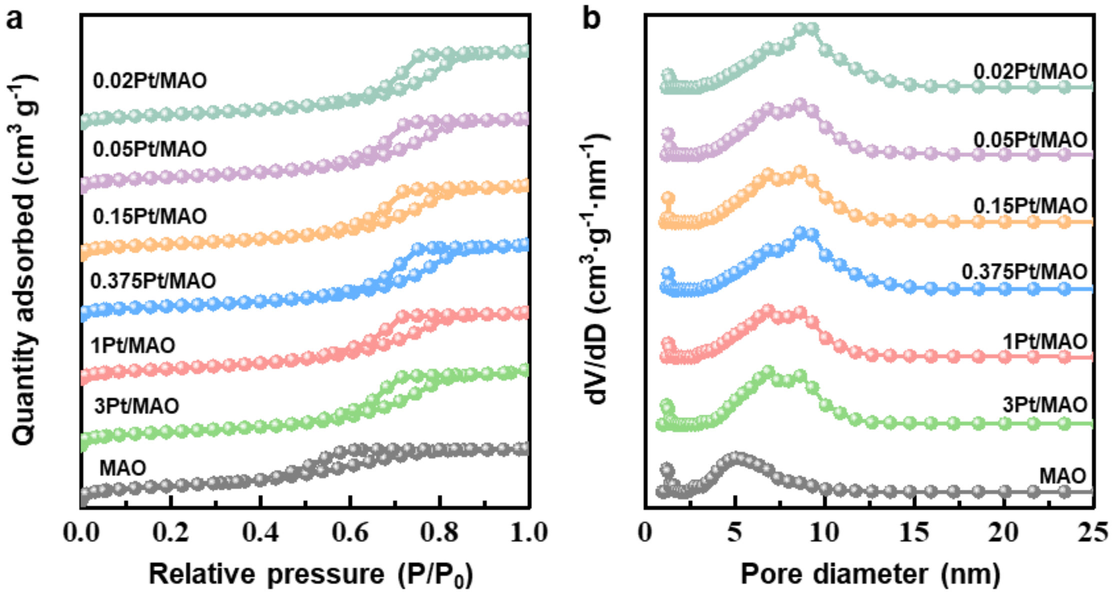

Figure S1. Textural structure characterization of the catalysts. (a) Nitrogen physisorption isotherms and (b) pore size distribution of Pt/MAO catalysts with varying Pt loadings.

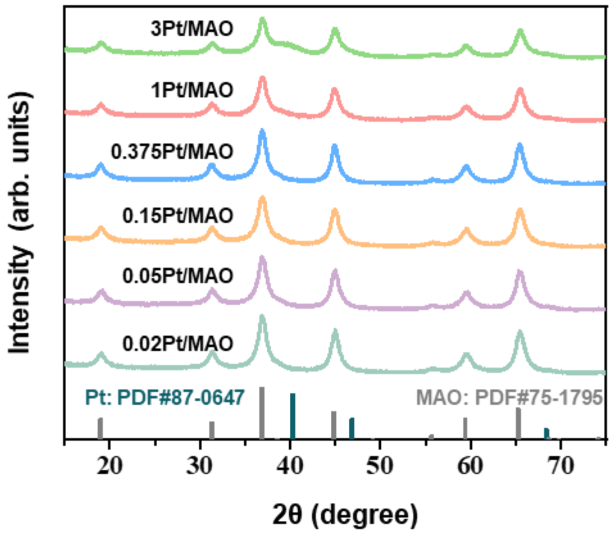

Figure S2. XRD characterization of the catalysts. XRD patterns of Pt/MAO catalysts with varying Pt loadings.

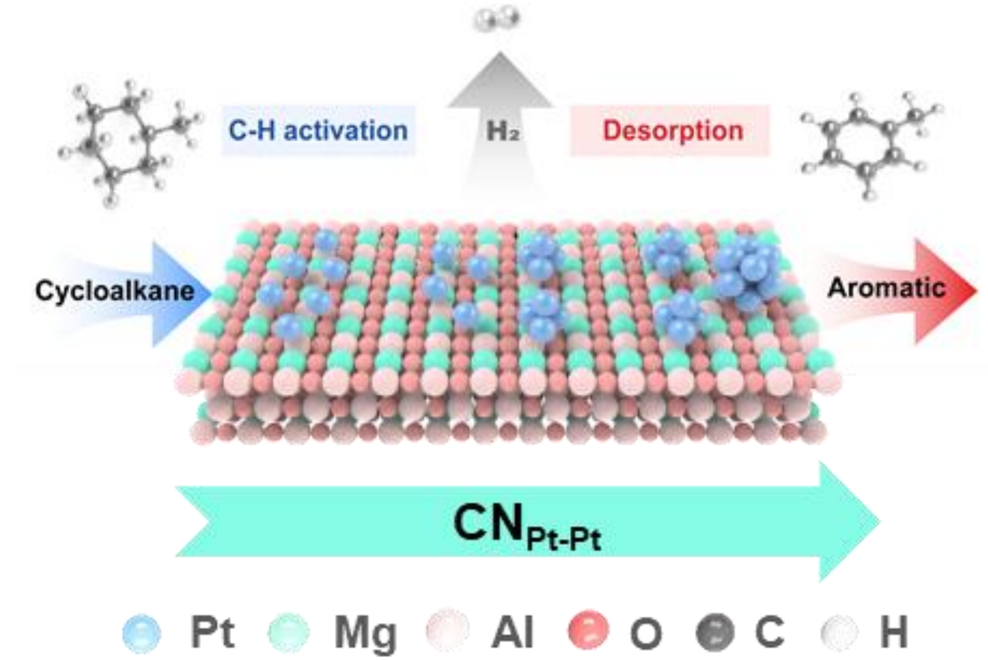

Figure S3. Schematic representation of Pt species distribution. Schematic representation the gradual growth in Pt species size with increasing Pt loading in Pt/MAO catalysts.

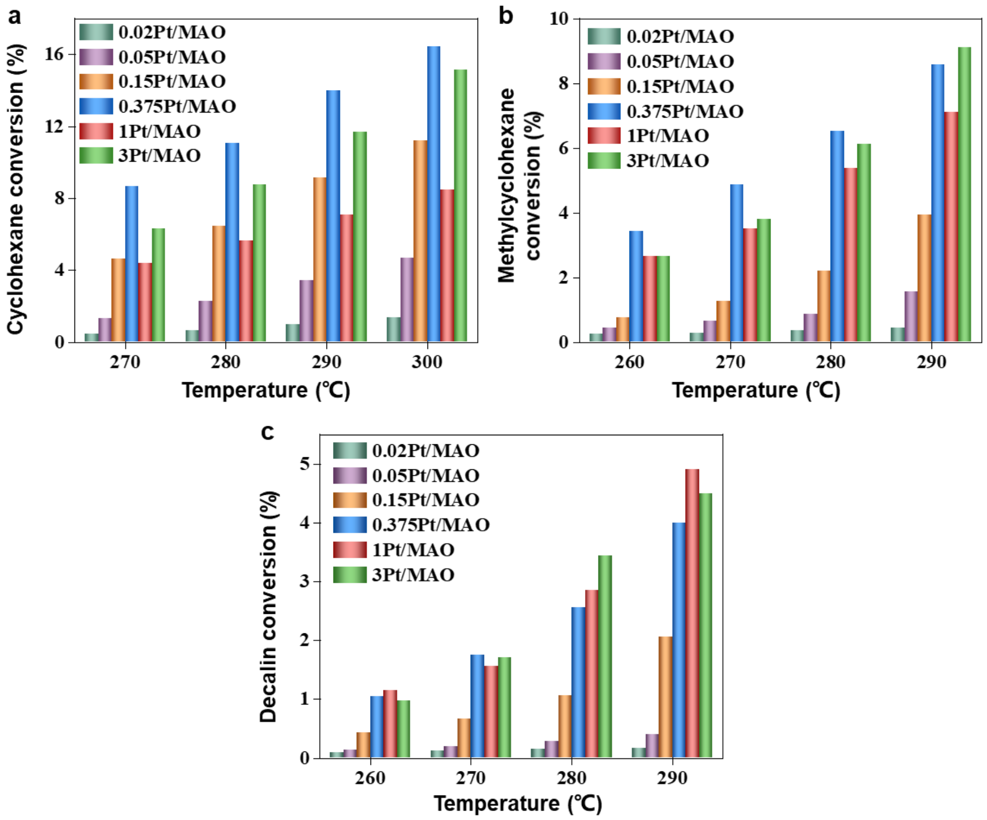

Figure S4. Catalytic performance of Pt/MAO catalysts toward the dehydrogenation of various cycloalkanes. The conversion of cyclohexane (a), methylcyclohexane (b) and decalin (c) on Pt/MAO catalysts with varying Pt loadings at different reaction temperatures.

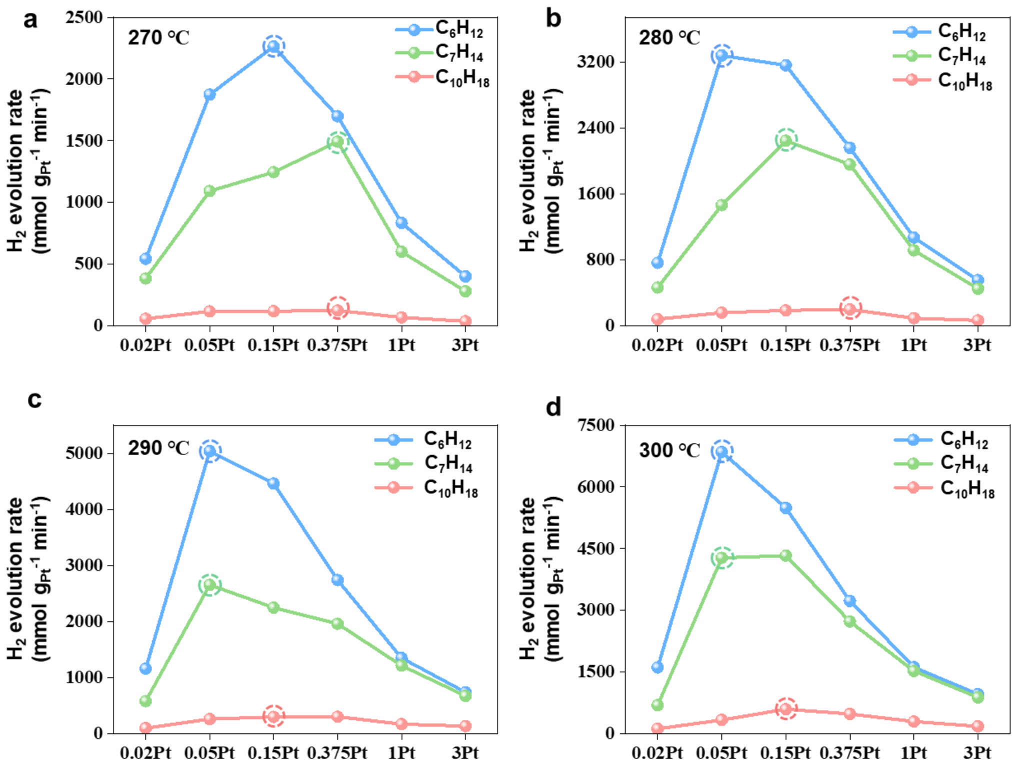

Figure S5. Temperature-dependent hydrogen release behavior of Pt/MAO catalysts toward the dehydrogenation of various cycloalkanes. Comparison of hydrogen evolution rates of Pt/MAO catalysts for the dehydrogenation of cyclohexane, methylcyclohexane, and decalin at different reaction temperatures: (a) 270 °C, (b) 280 °C, (c) 290 °C, and (d) 300 °C.

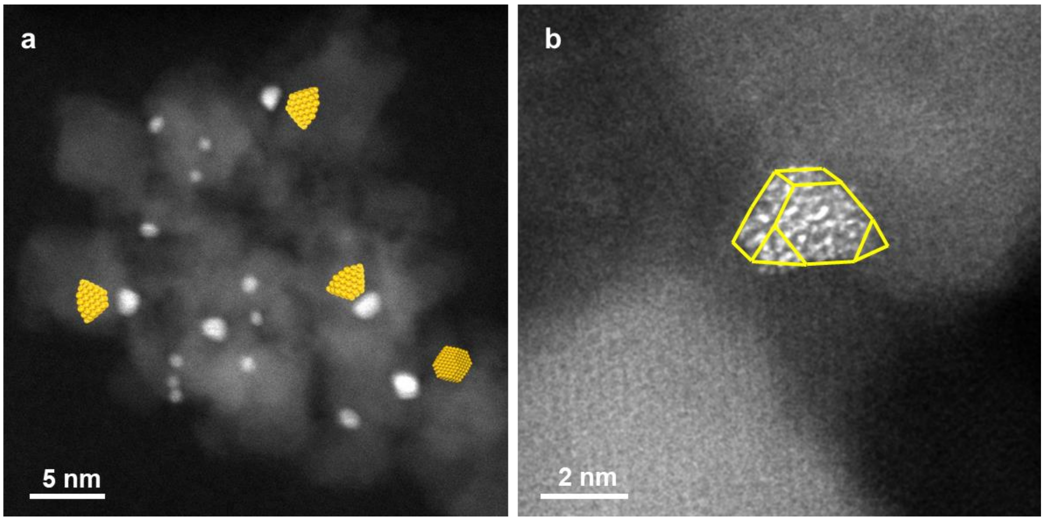

Figure S6. Atomic-scale morphology of Pt nanoparticles supported on MAO.

HAADF-STEM images of Pt nanoparticles in 1Pt/MAO catalysts with schematic truncated octahedral models overlaid.

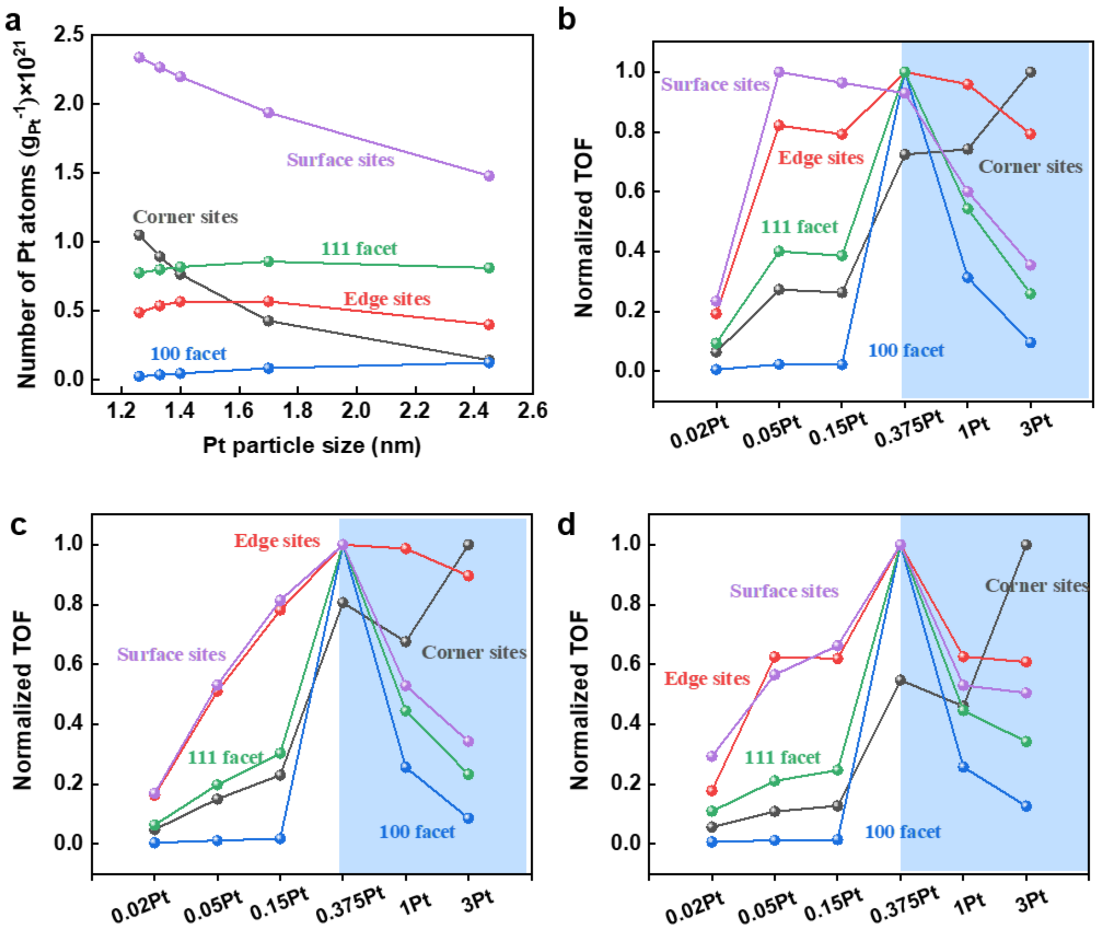

Figure S7. Correlation between Pt nanoparticle morphology and intrinsic catalytic activity in cycloalkane dehydrogenation. (a) Number of surface atoms per gram of Pt as a function of Pt particle size based on a truncated octahedral model. (b-d) Normalized TOFs calculated from hydrogen evolution rates for (b) cyclohexane dehydrogenation, (c) methylcyclohexane dehydrogenation, and (d) decalin dehydrogenation.

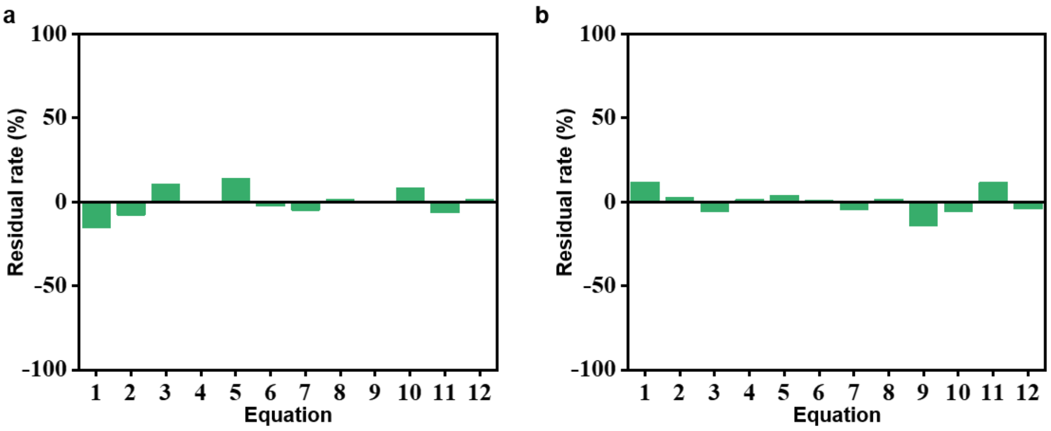

Figure S8. Residuals of least-squares fitting for Pt species quantification.

Residual rates from least-squares fitting for determining the fractions of Pt species in (a) 0.05Pt/MAO and (b) 0.15Pt/MAO catalysts.

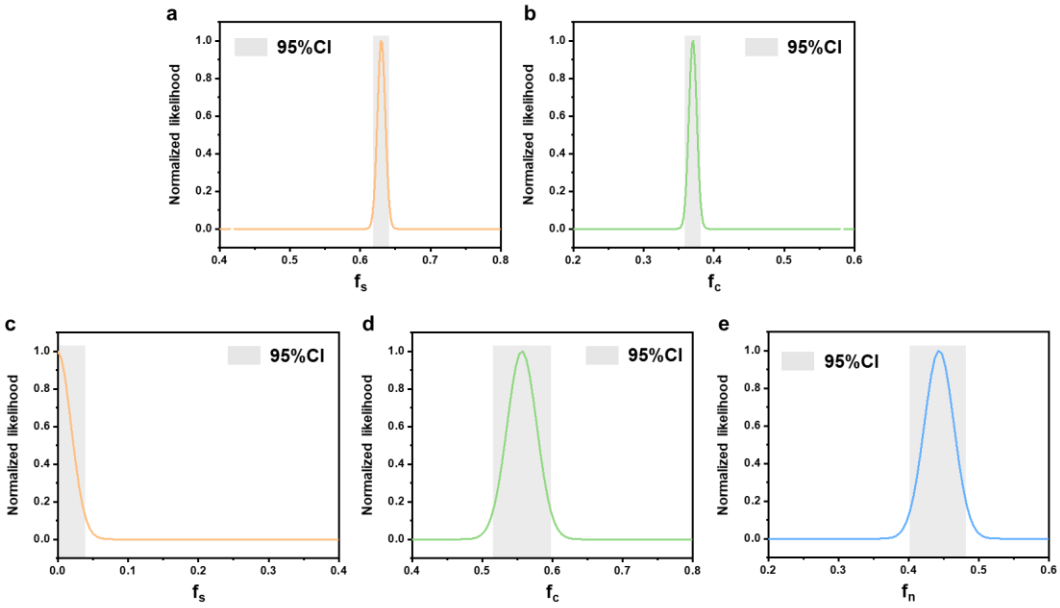

Figure S9. Statistical confidence analysis of fitted Pt species fractions. Normalized likelihood curves for fitted site fractions of Pt single atom (f_s), Pt cluster (f_c), and Pt nanoparticle (f_n) in 0.05Pt/MAO (a-b) and 0.15Pt/MAO (c-e) catalysts. Gray shaded regions indicate 95% confidence intervals based on the likelihood threshold criterion.

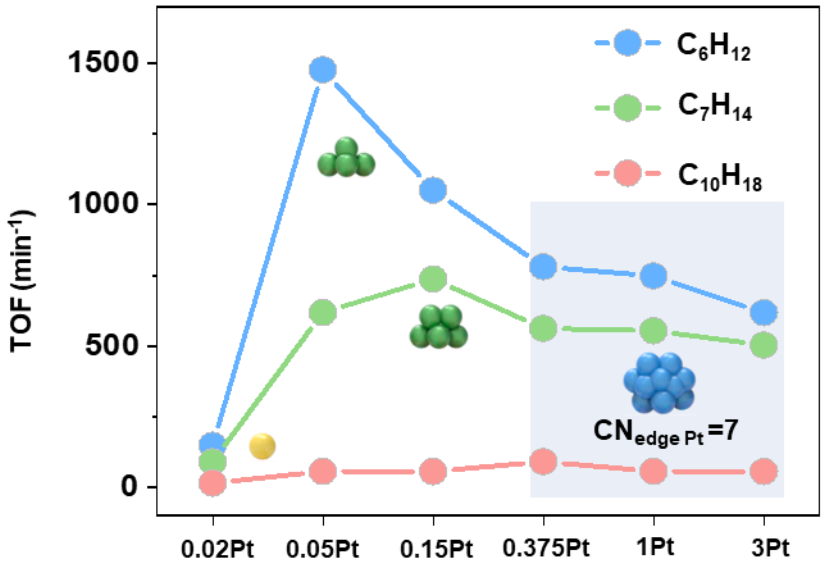

Figure S10. Site-specific TOFs of Pt/MAO catalysts. Site-specific TOF of Pt/MAO catalysts for different dehydrogenation reactions at 280 °C.

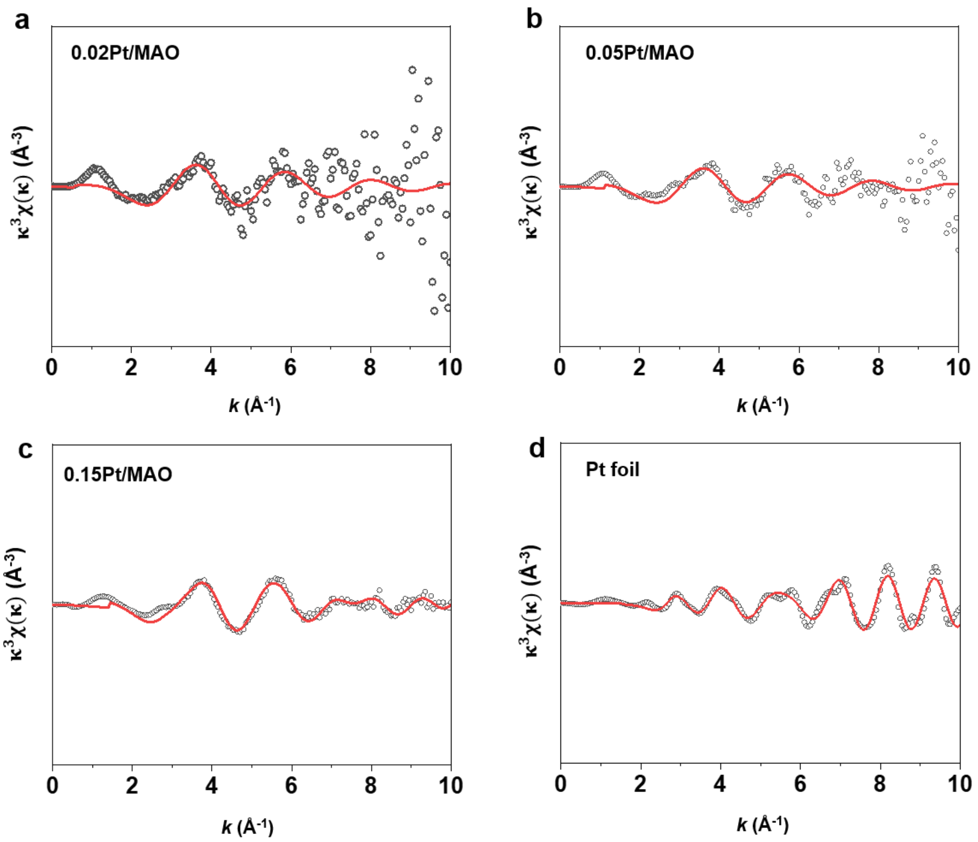

Figure S11. EXAFS fitting of Pt L$_{III}$-edge spectra in k-space. K-space EXAFS spectra and corresponding fitting curves for (a) 0.02Pt/MAO, (b) 0.05Pt/MAO, (c) 0.15Pt/MAO, and (d) Pt foil samples. Black dots and red line represent the original and fitting results, respectively.

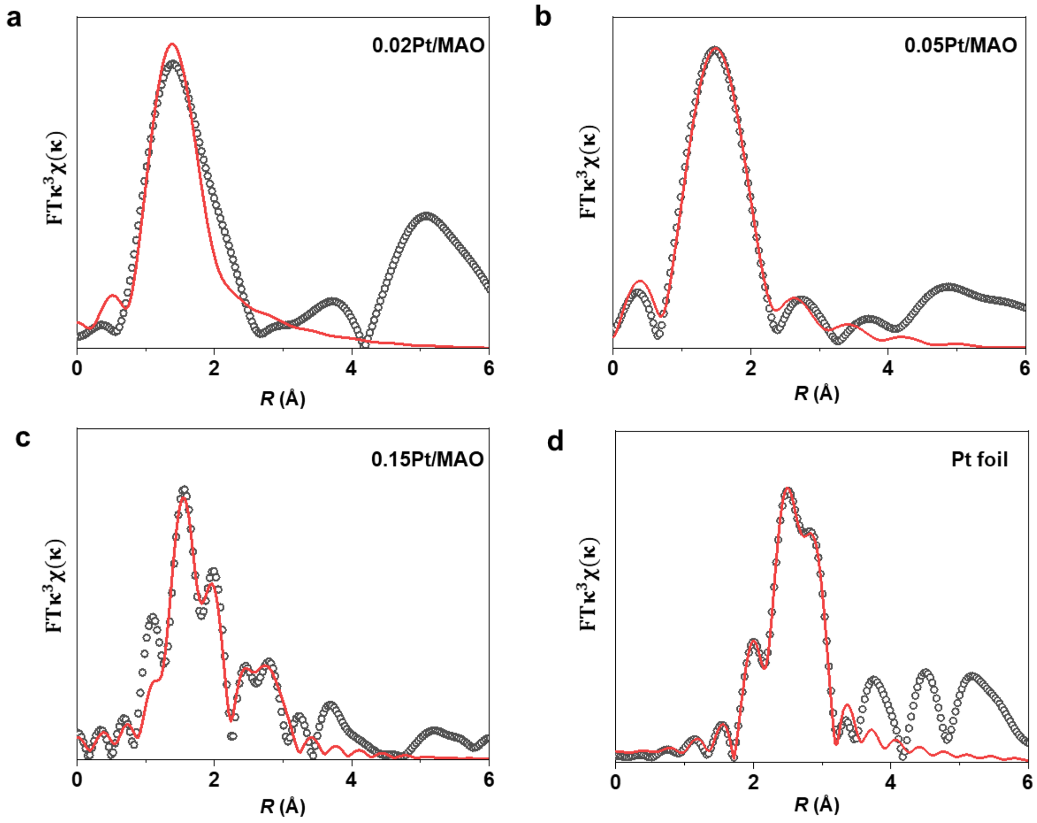

Figure S12. EXAFS fitting of Pt L$_{III}$-edge spectra in R-space. R-space EXAFS spectra and corresponding fitting curves for (a) 0.02Pt/MAO, (b) 0.05Pt/MAO, (c) 0.15Pt/MAO, and (d) Pt foil samples. Black dots and red line represent the original and fitting results, respectively.

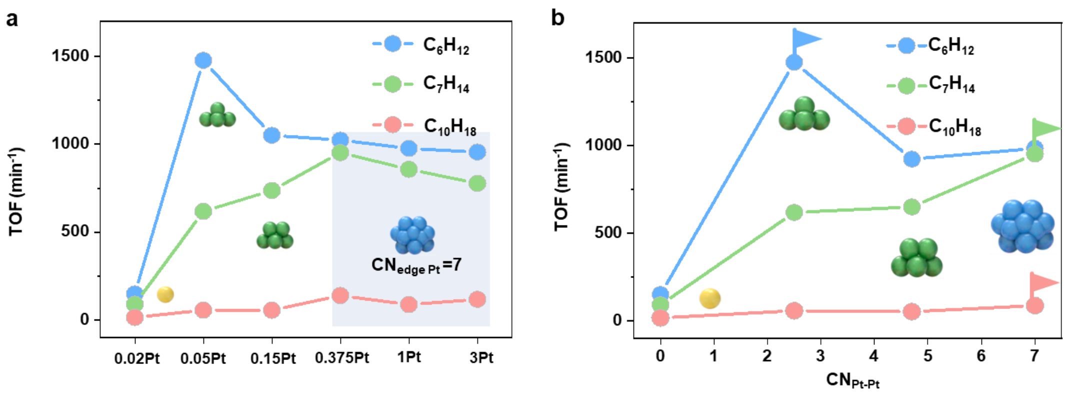

Figure S13. Site-specific TOF of Pt/MAO catalysts estimated using the cuboctahedron model. (a) TOF across different Pt loadings and (b) TOF as a function of CN_{Pt-Pt} for different dehydrogenation reactions at 280 °C.

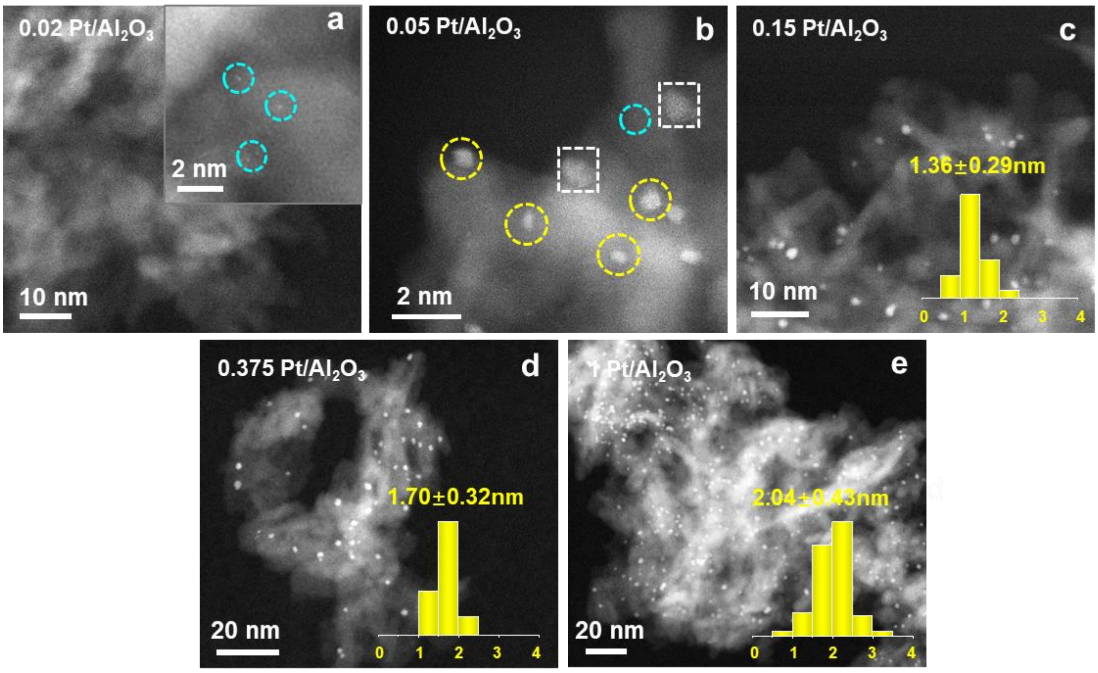

Figure S14. HAADF-STEM images and particle size distributions of Pt/Al_2O_3 catalysts. (a) 0.02Pt/Al_2O_3, (b) 0.05Pt/Al_2O_3, (c) 0.15Pt/Al_2O_3, (d) 0.375Pt/Al_2O_3, and (e) 1Pt/Al_2O_3 catalysts.

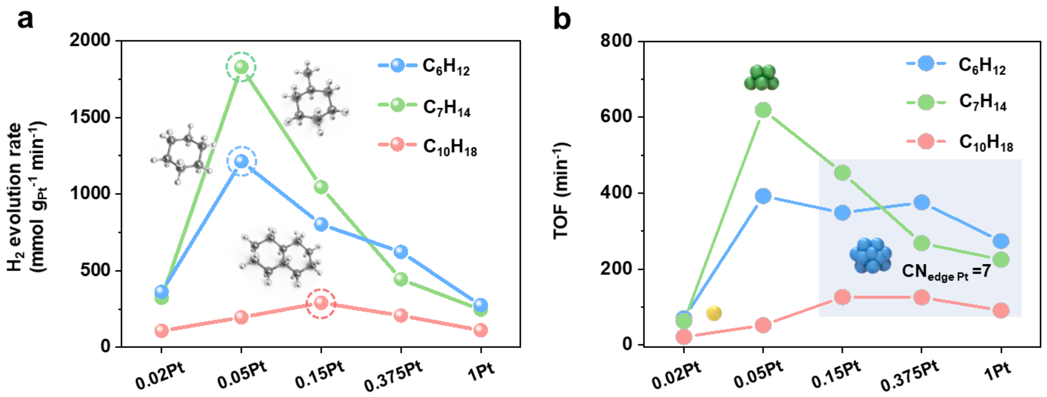

Figure S15. Hydrogen evolution activities of Pt/Al_2O_3 catalysts. (a) Hydrogen evolution rates and (b) site-specific TOFs of Pt/Al_2O_3 catalysts for the dehydrogenation of cyclohexane, methylcyclohexane, and decalin at 280 °C.

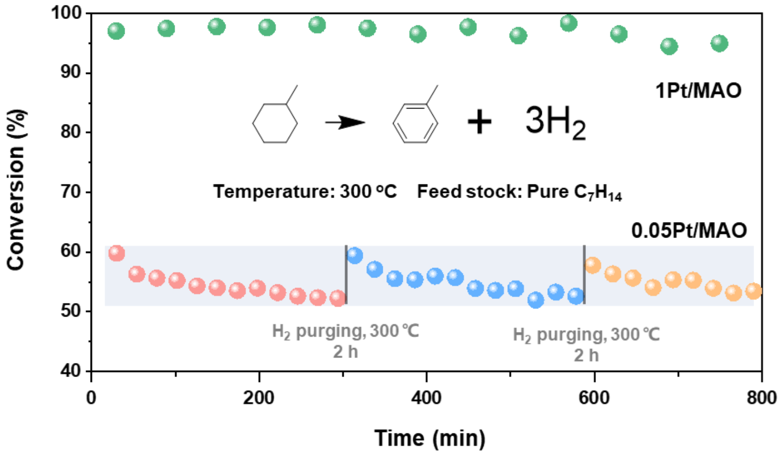

Figure S16. Long-term stability of Pt/MAO catalysts. Long-term stability of 1Pt/MAO and 0.05Pt/MAO for methylcyclohexane dehydrogenation at 300 °C. Activity loss on 0.05Pt/MAO is reversible upon H_2 purging.

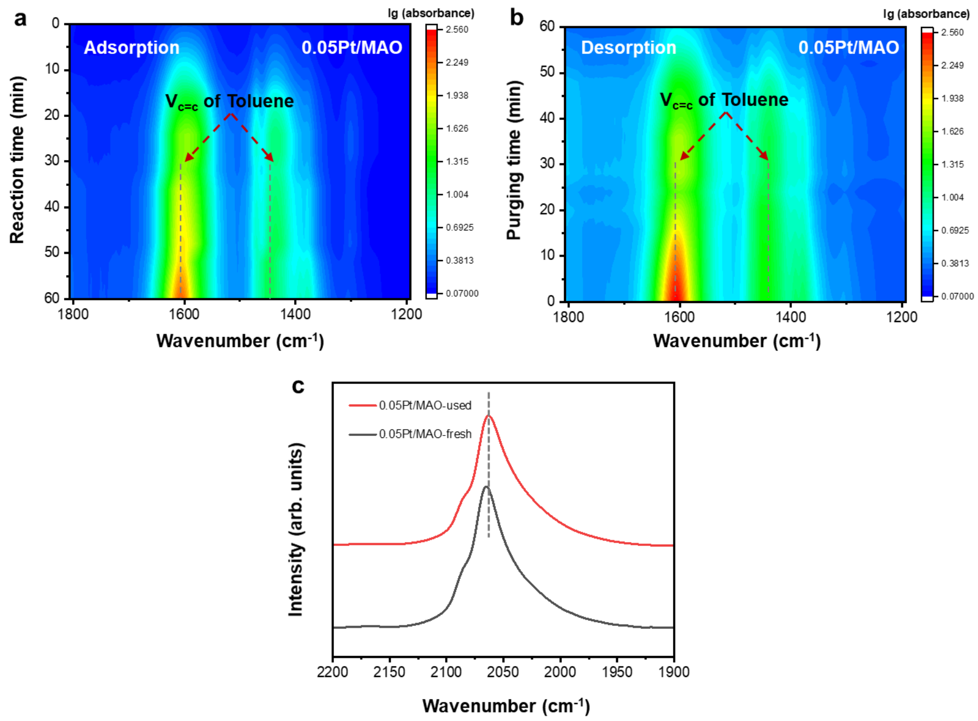

Figure S17. In-situ FTIR and CO-DRIFTS characterization of 0.05Pt/MAO catalyst. (a) In situ FTIR spectra collected during methylcyclohexane dehydrogenation. (b) Corresponding spectra during subsequent H_2 purging. (c) CO-DRIFTS spectra of 0.05Pt/MAO before and after long-term testing.

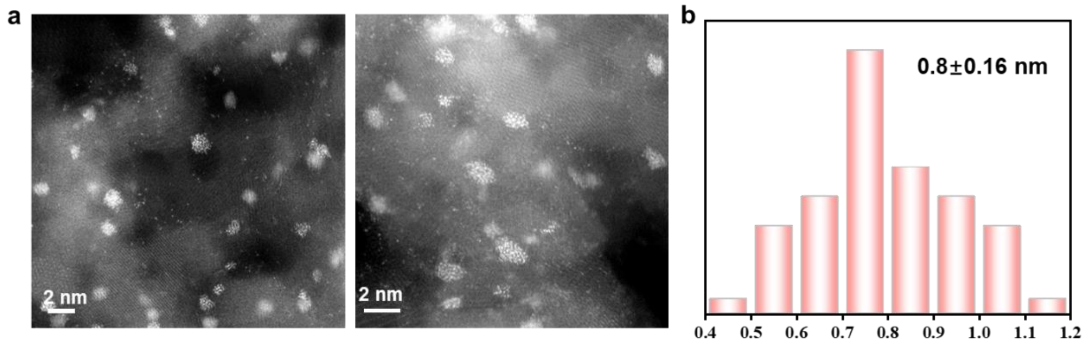

Figure S18. Structural characterization of spent 0.15Pt/MAO catalyst. HAADF-STEM images (a) and particle size distribution (b) of spent 0.15Pt/MAO catalyst after 100 h dehydrogenation.

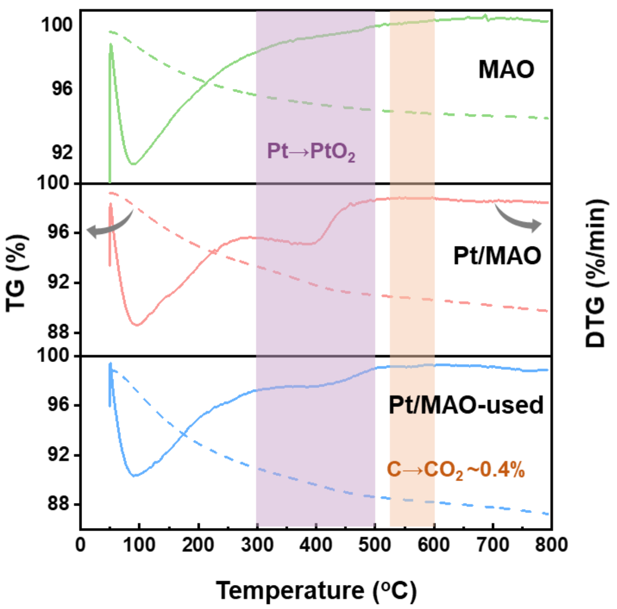

Figure S19. Coke resistance characterization of Pt catalysts. TG profiles of MAO, fresh 0.15Pt/MAO, and spent 0.15Pt/MAO samples.

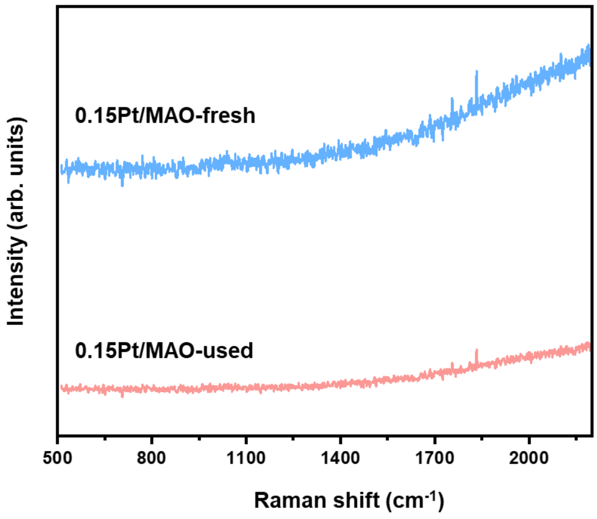

Figure S20. Coke characterization of Pt/MAO catalyst. Raman spectroscopy of 0.15Pt/MAO catalyst before and after long-term testing.

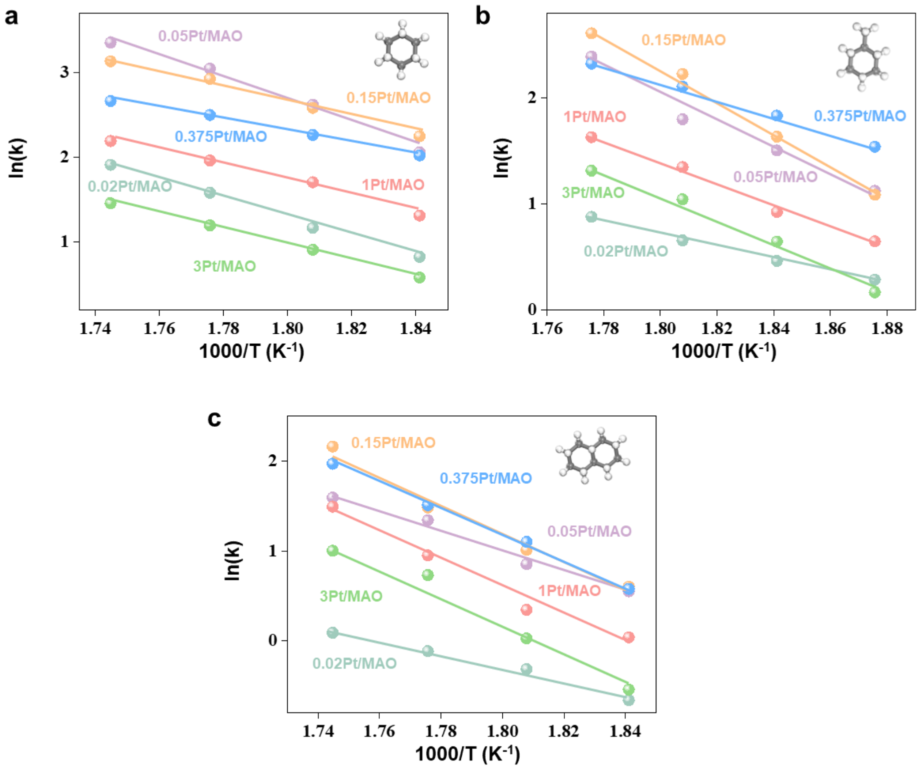

Figure S21. Arrhenius plots of Pt/MAO catalysts. Arrhenius plots for calculation of activation energy over various Pt/MAO catalysts for (a) cyclohexane dehydrogenation, (b) methylcyclohexane dehydrogenation, and (c) decalin dehydrogenation.

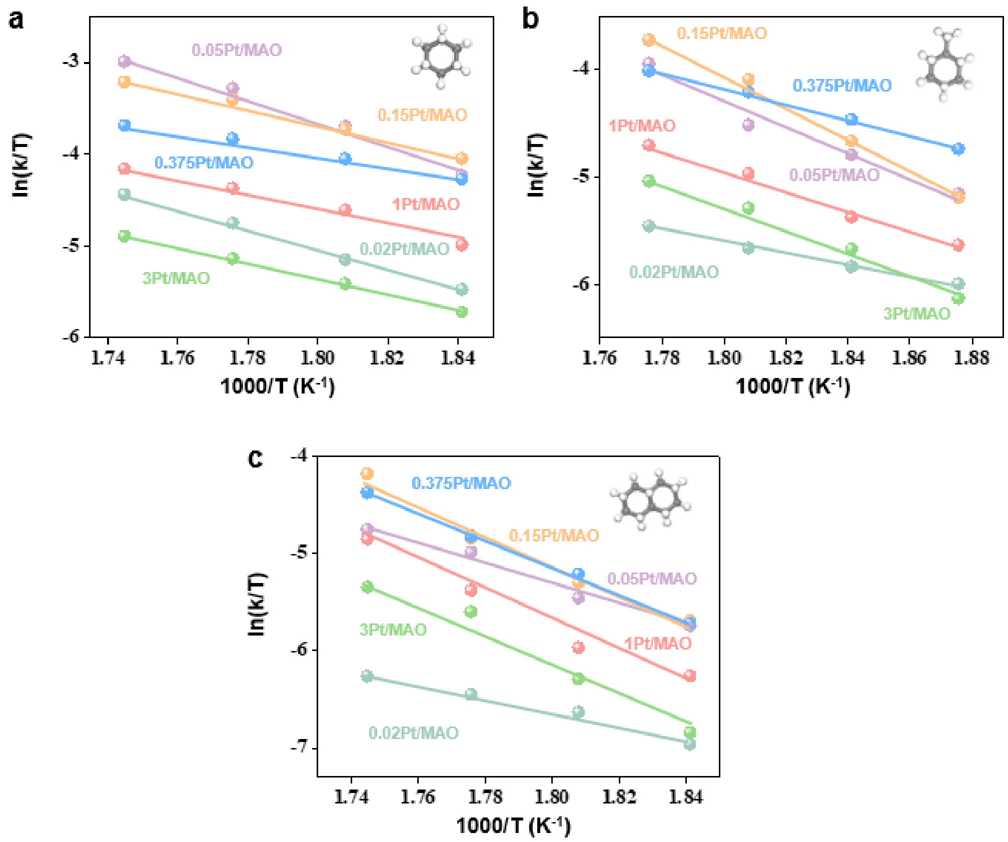

Figure S22. Eyring plots of Pt/MAO catalysts. Eyring plots for calculation of entropy change over various Pt/MAO catalysts for (a) cyclohexane dehydrogenation, (b) methylcyclohexane dehydrogenation, and (c) decalin dehydrogenation.

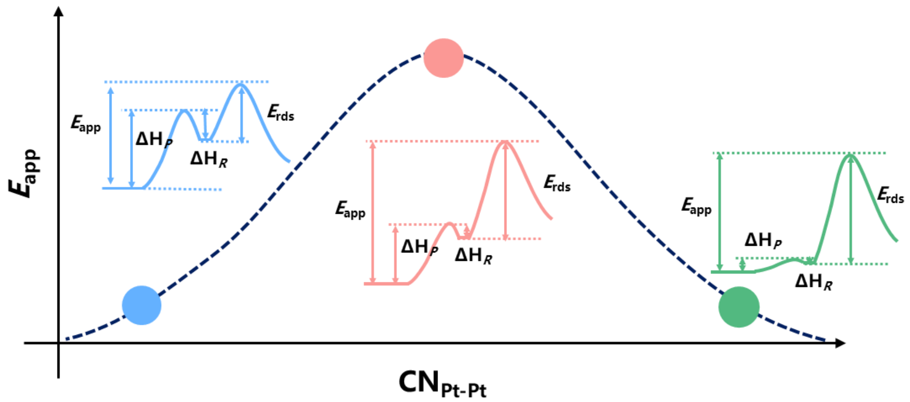

Figure S23. Relationship between Pt coordination and apparent activation energy. Correlation between Pt coordination number and E_{app} governed by adsorption-desorption thermodynamics in Pt/MAO catalysts.

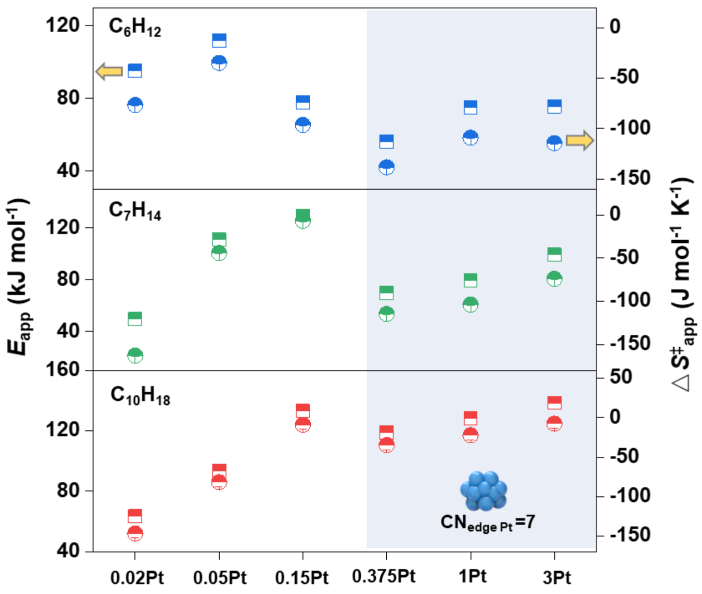

Figure S24. Entropic and enthalpic trends in dehydrogenation on Pt/MAO catalysts. Apparent activation energy (square) and entropy (circle) of Pt/MAO catalysts for the dehydrogenation of cyclohexane, methylcyclohexane and decalin. Error bars of $\Delta S^{\ddagger}$ reflect uncertainties derived from ±50% variation in the assumed reaction order.

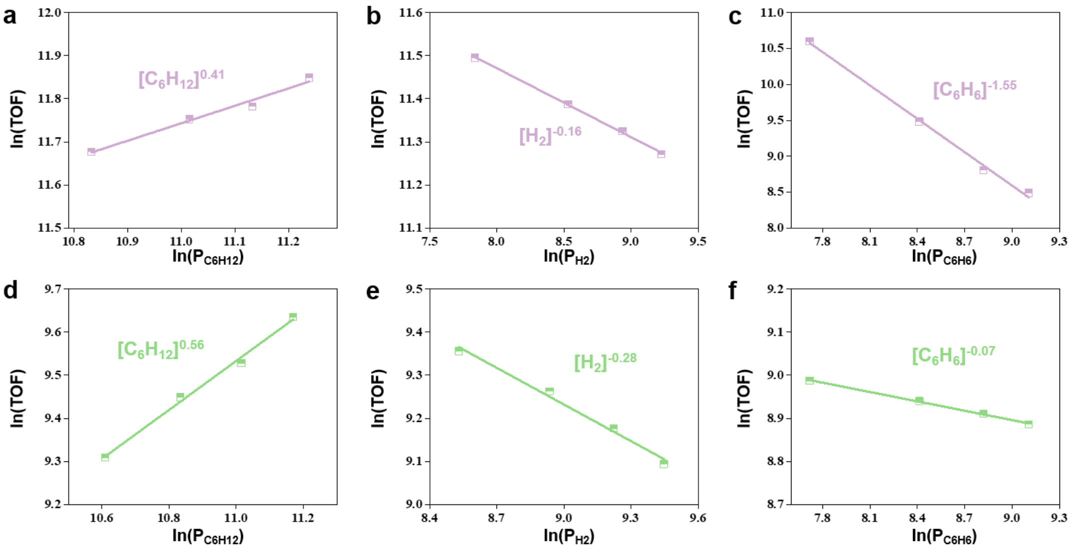

Figure S25. Kinetic dependence of reaction rates on partial pressures for cyclohexane dehydrogenation. Correlation between partial pressures of (a, d) C_6H_{12}, (b, e) H_2, and (c, f) C_6H_6 with reaction rates over 0.05Pt/MAO and 3Pt/MAO catalysts.

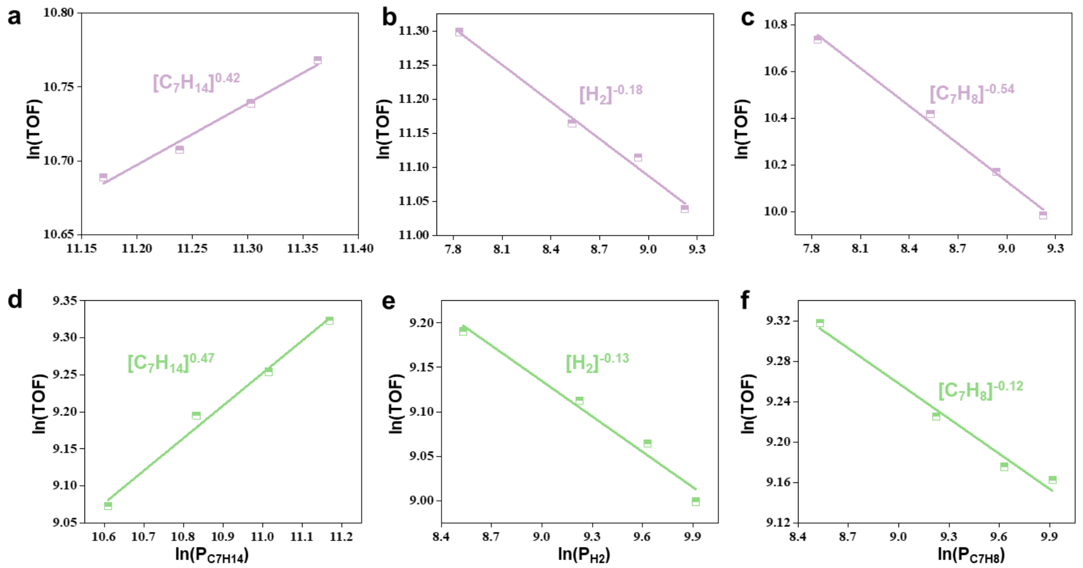

Figure S26. Kinetic dependence of reaction rates on partial pressures for methylcyclohexane dehydrogenation. Correlation between partial pressures of (a, d) C_7H_{14}, (b, e) H_2, and (c, f) C_7H_8 with reaction rates over 0.05Pt/MAO and 3Pt/MAO catalysts.

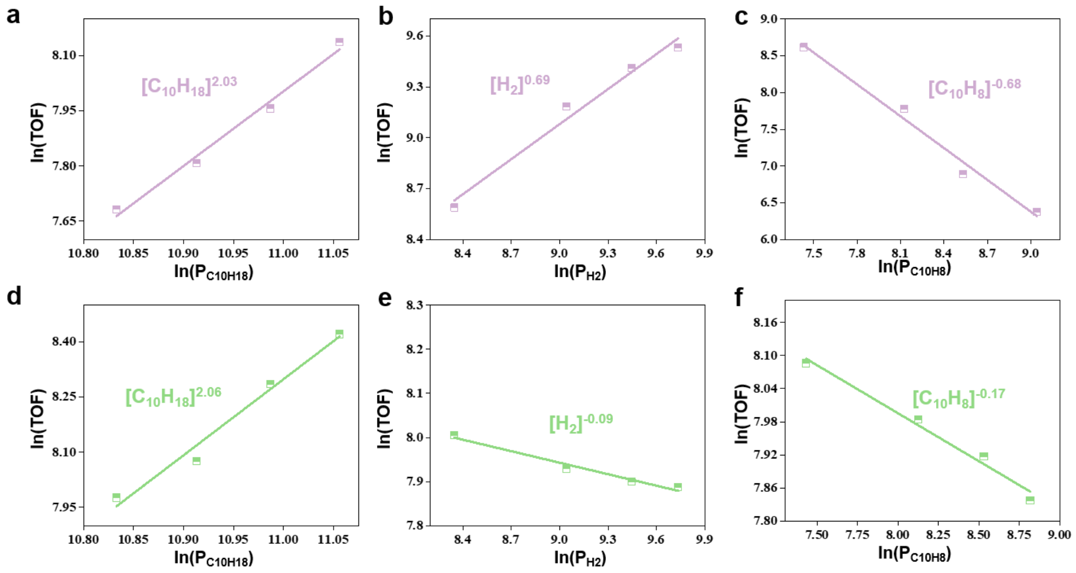

Figure S27. Kinetic dependence of reaction rates on partial pressures for decalin dehydrogenation. Correlation between partial pressures of (a, d) $C_{10}H_{18}$, (b, e) H_2, and (c, f) $C_{10}H_8$ with reaction rates over 0.05Pt/MAO and 3Pt/MAO catalysts.

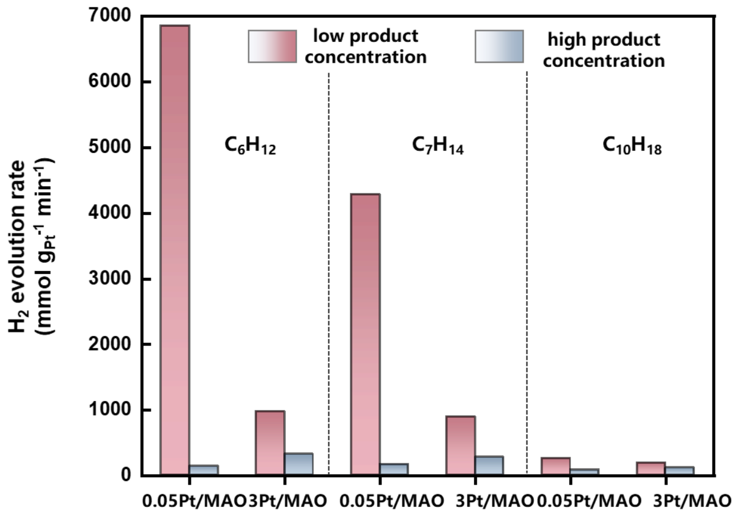

Figure S28. Product inhibition effects in dehydrogenation. Dehydrogenation rates of cyclohexane, methylcyclohexane, and decalin over 0.05Pt/MAO and 3Pt/MAO catalysts at 300 ^{o}C under low (0) and high (0.5) product partial pressures.

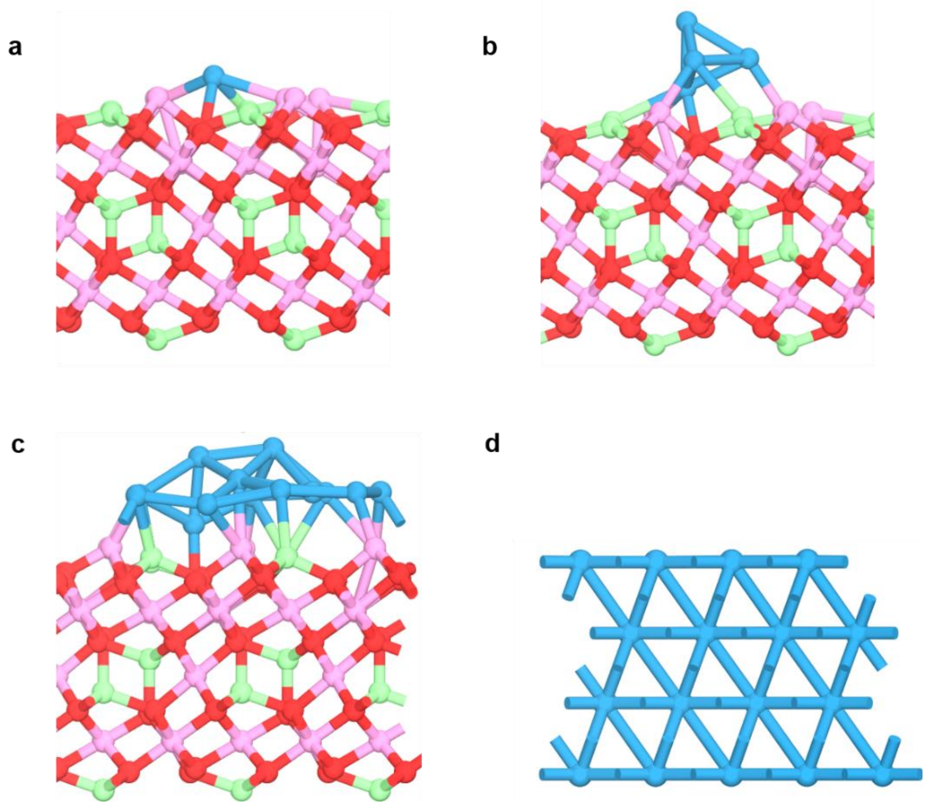

Figure S29. Structural models of Pt species on the MAO support. Optimized geometries of (a) Pt_1/MAO, (b) Pt_4/MAO, (c) Pt_{13}/MAO and (d) Pt(111). Color code: Pt, blue; O, red; Al, pink; Mg, green.

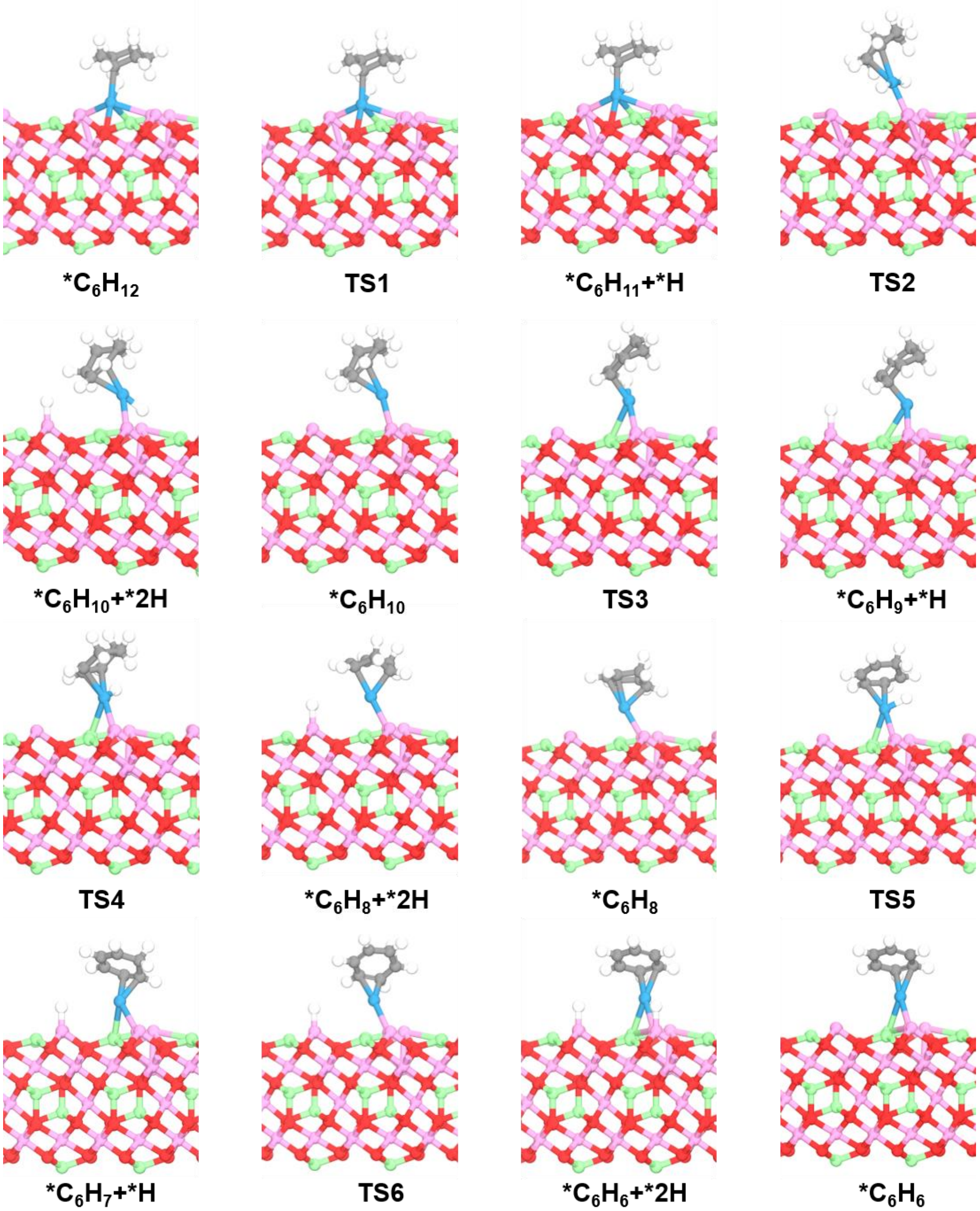

Figure S30. DFT-optimized geometries for cyclohexane dehydrogenation on Pt_1/MAO. Adsorption configuration diagram of cyclohexane dehydrogenation on Pt_1/MAO. Color code: Pt, blue; O, red; Al, pink; Mg, green; C, gray; H, white.

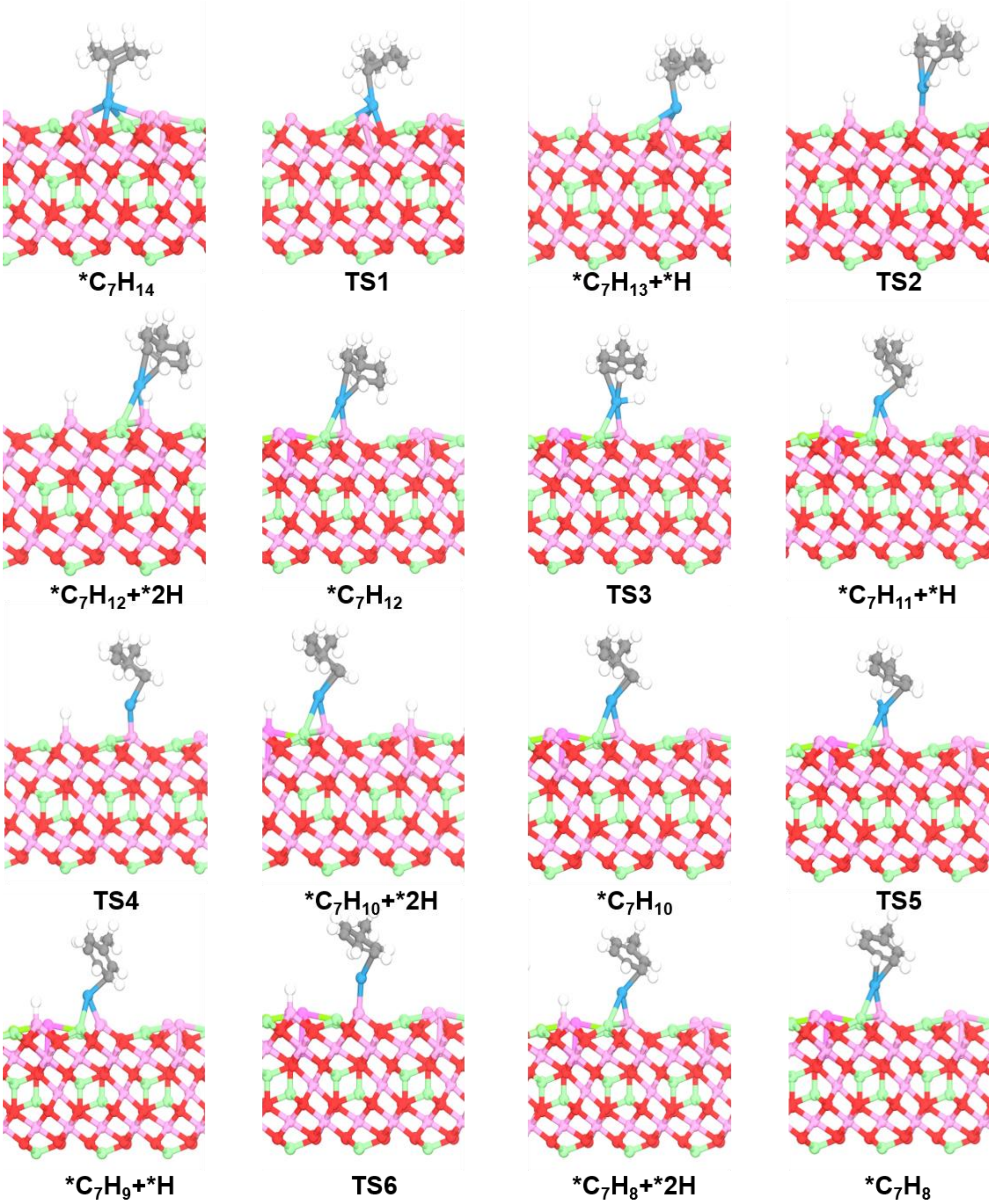

Figure S31. DFT-optimized geometries for methylcyclohexane dehydrogenation on Pt_1/MAO. Adsorption configuration diagram of methylcyclohexane dehydrogenation on Pt_1/MAO. Color code: Pt, blue; O, red; Al, pink; Mg, green; C, gray; H, white.

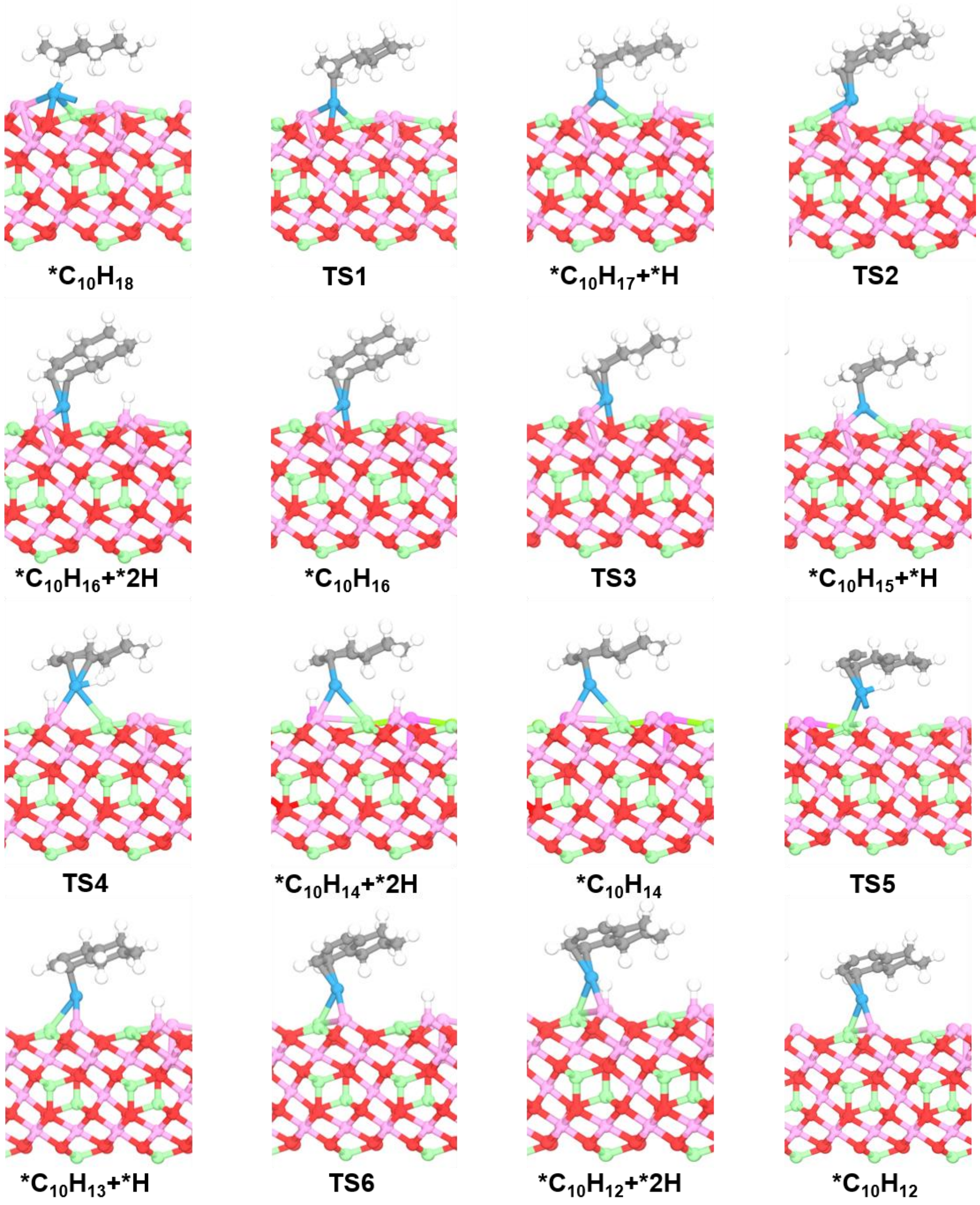

Figure S32. DFT-optimized geometries for decalin dehydrogenation on Pt_1/MAO.

Adsorption configuration diagram of decalin dehydrogenation on Pt_1/MAO. Color code: Pt, blue; O, red; Al, pink; Mg, green; C, gray; H, white.

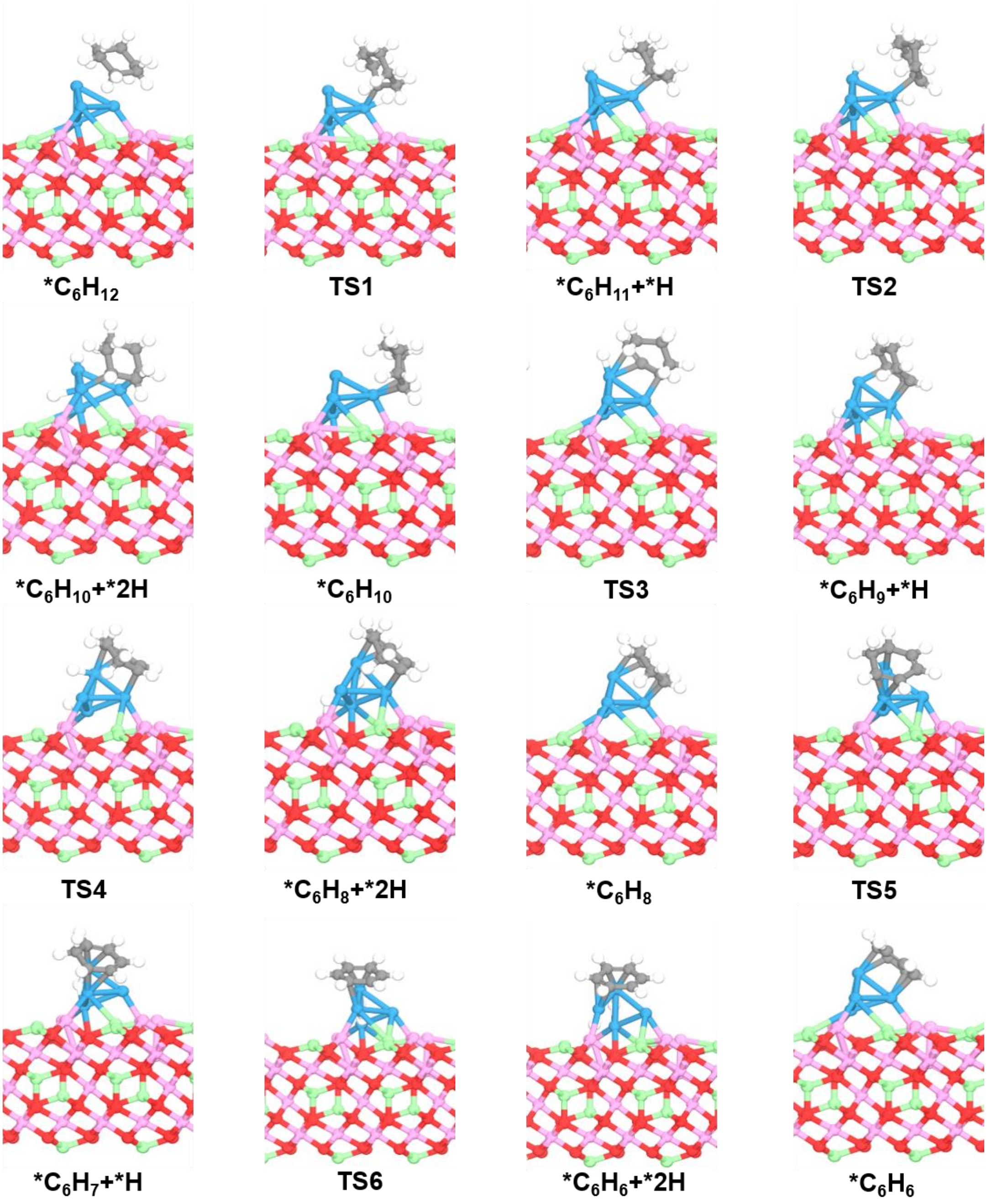

Figure S33. DFT-optimized geometries for cyclohexane dehydrogenation on Pt_4/MAO. Adsorption configuration diagram of cyclohexane dehydrogenation on Pt_4/MAO. Color code: Pt, blue; O, red; Al, pink; Mg, green; C, gray; H, white.

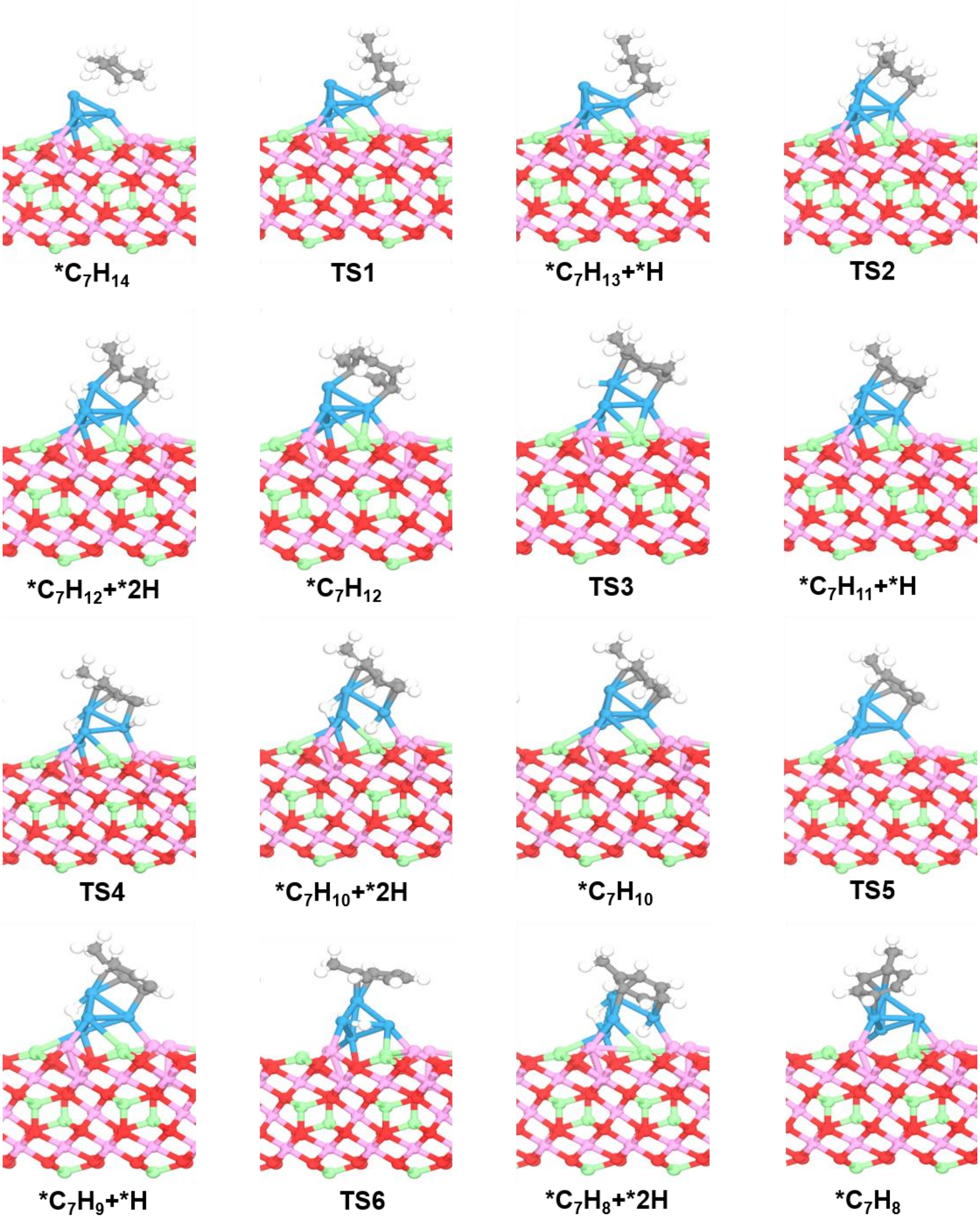

Figure S34. DFT-optimized geometries for methylcyclohexane dehydrogenation on Pt_4/MAO. Adsorption configuration diagram of methylcyclohexane dehydrogenation on Pt_4/MAO. Color code: Pt, blue; O, red; Al, pink; Mg, green; C, gray; H, white.

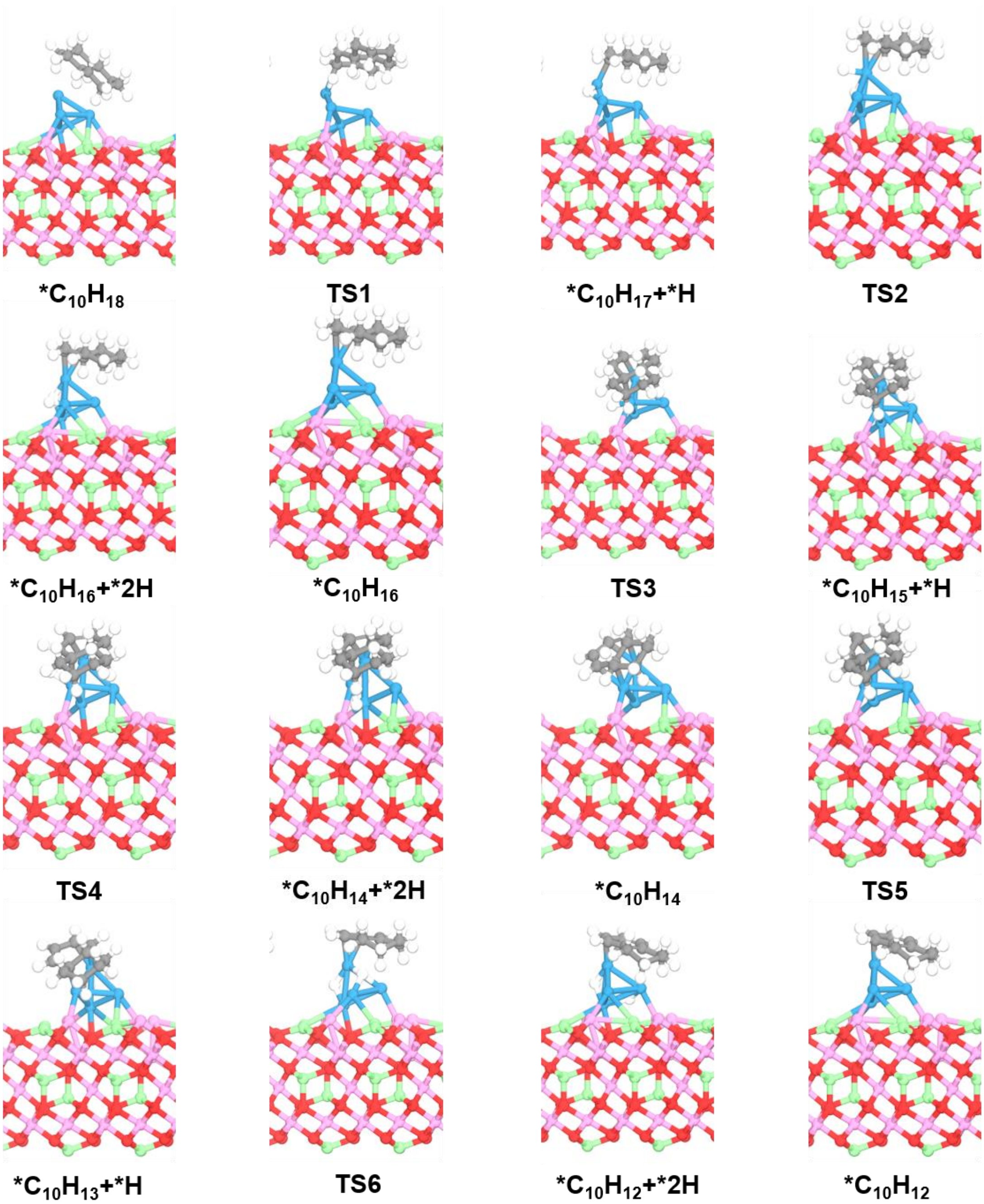

Figure S35. DFT-optimized geometries for decalin dehydrogenation on Pt_4/MAO.

Adsorption configuration diagram of decalin dehydrogenation on Pt_4/MAO. Color code: Pt, blue; O, red; Al, pink; Mg, green; C, gray; H, white.

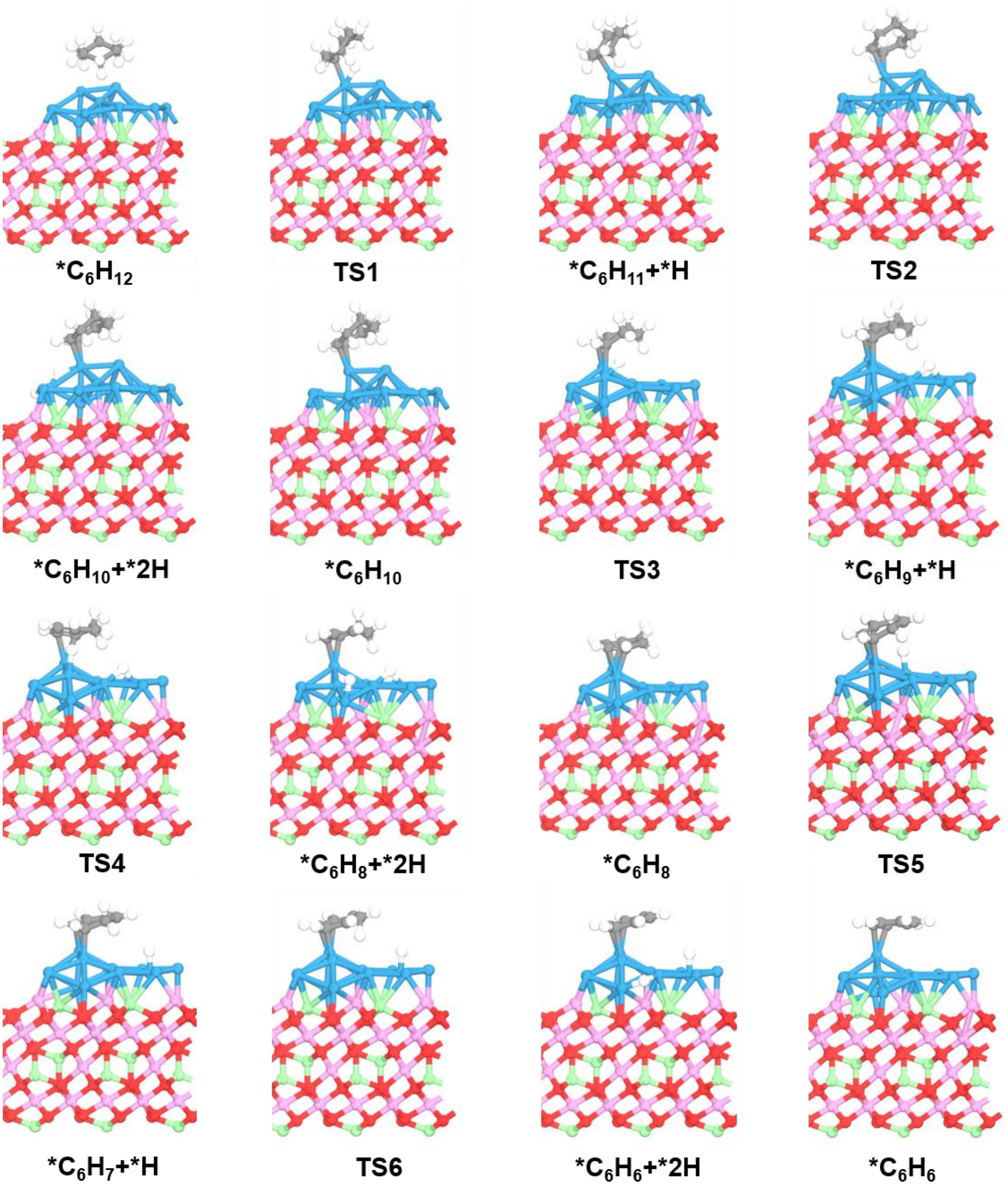

Figure S36. DFT-optimized geometries for cyclohexane dehydrogenation on Pt_{13}/MAO. Adsorption configuration diagram of cyclohexane dehydrogenation on Pt_{13}/MAO. Color code: Pt, blue; O, red; Al, pink; Mg, green; C, gray; H, white.

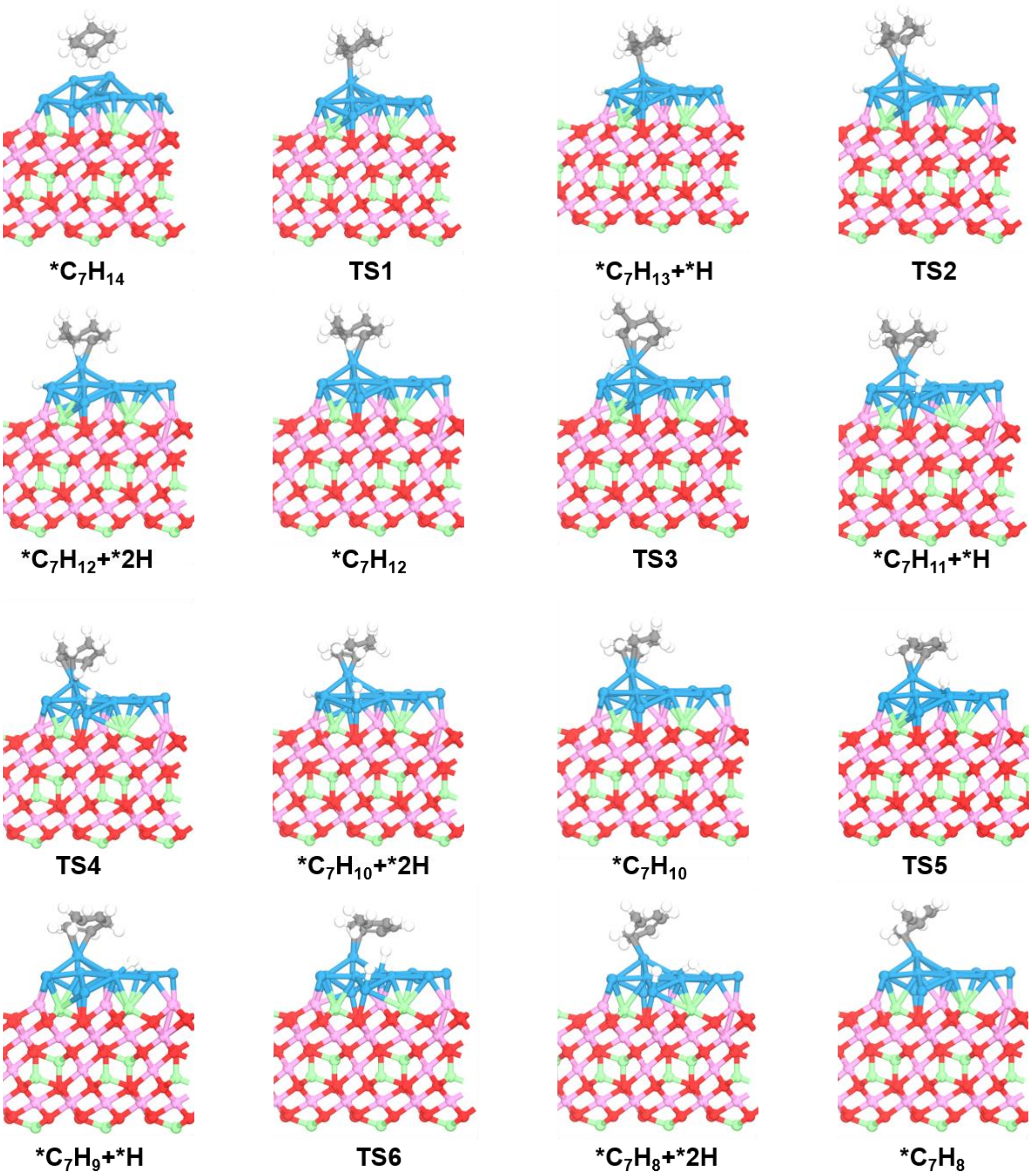

Figure S37. DFT-optimized geometries for methylcyclohexane dehydrogenation on Pt_{13}/MAO. Adsorption configuration diagram of methylcyclohexane dehydrogenation on Pt_{13}/MAO. Color code: Pt, blue; O, red; Al, pink; Mg, green; C, gray; H, white.

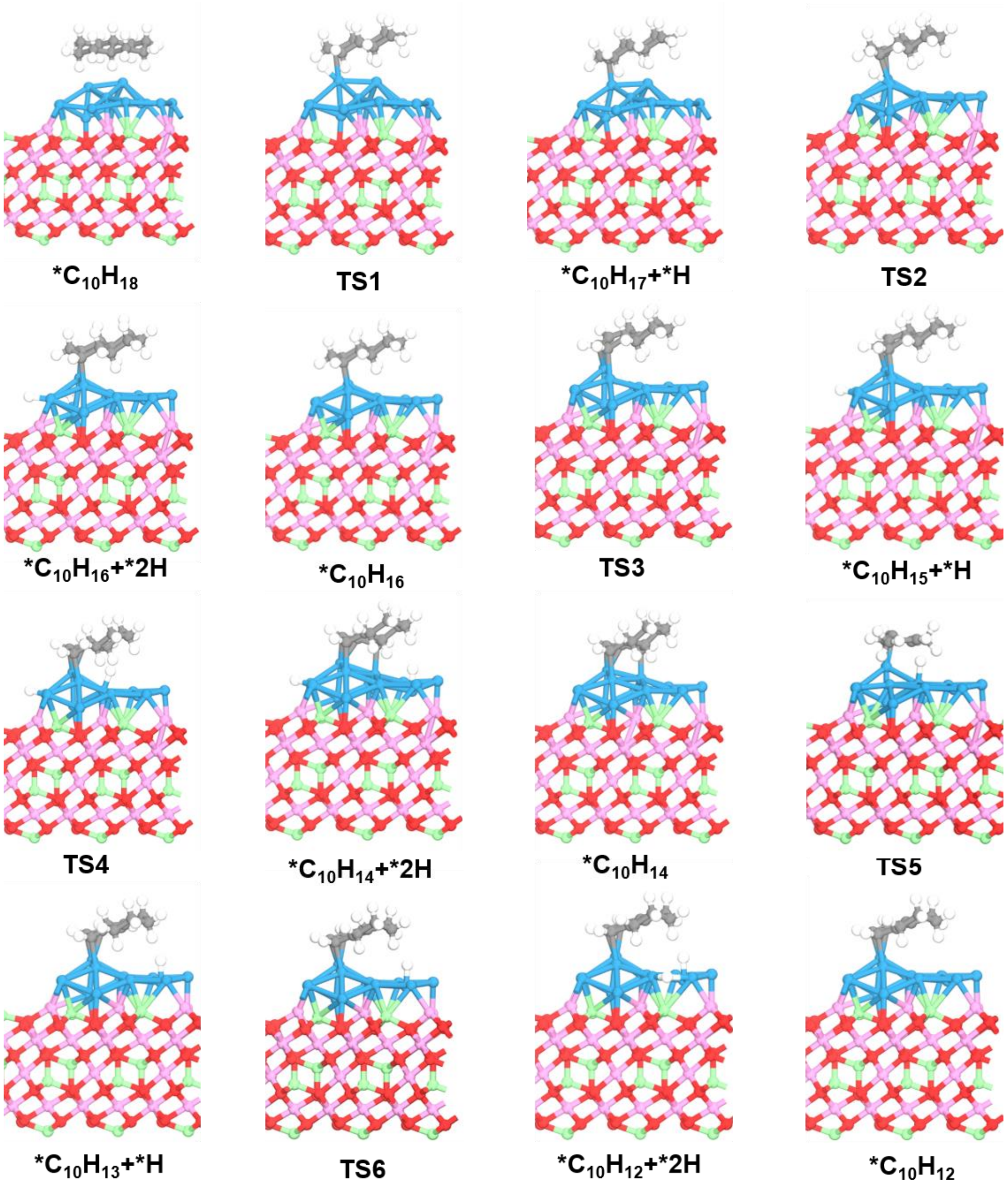

Figure S38. DFT-optimized geometries for decalin dehydrogenation on Pt_{13}/MAO. Adsorption configuration diagram of decalin dehydrogenation on Pt_{13}/MAO. Color code: Pt, blue; O, red; Al, pink; Mg, green; C, gray; H, white.

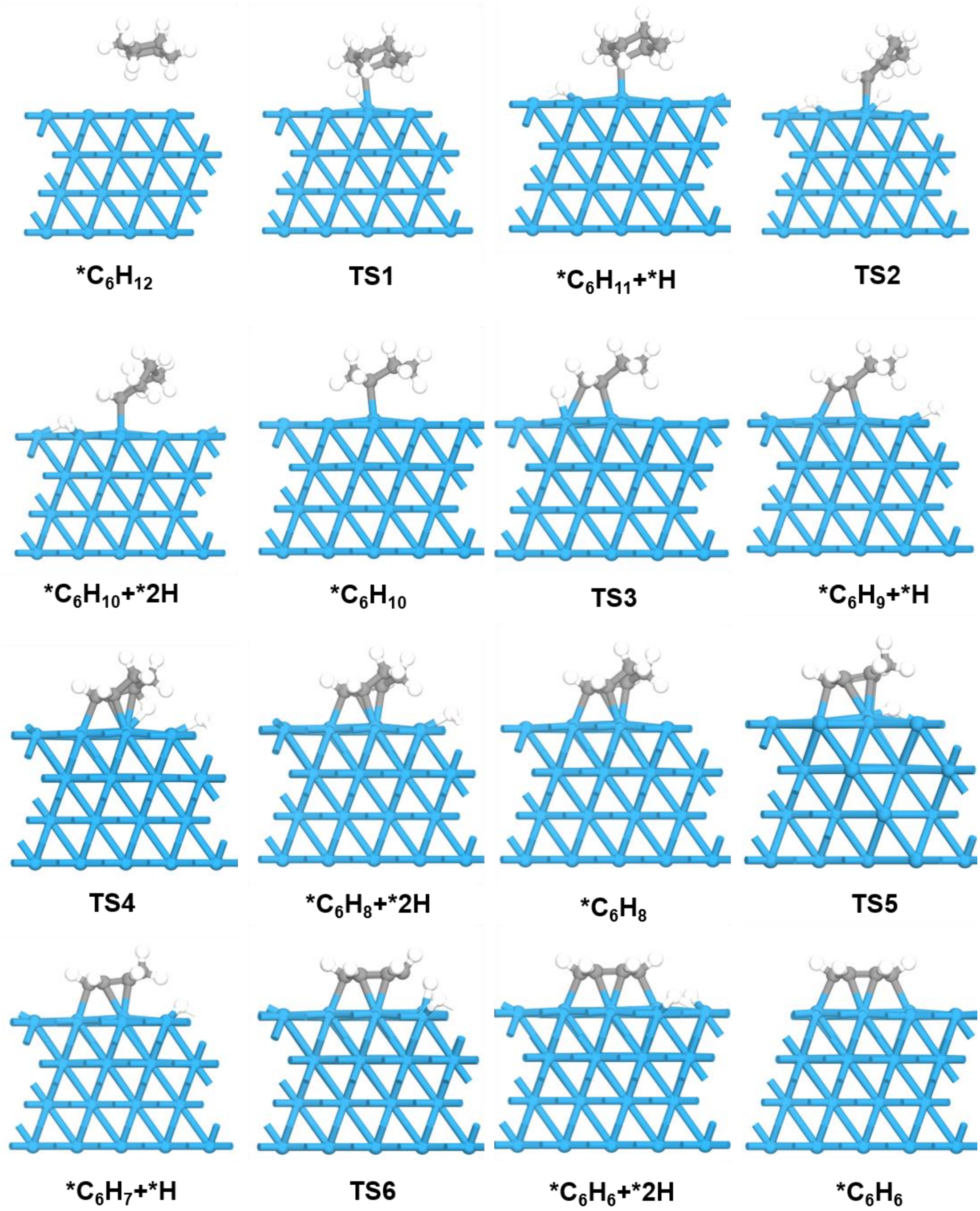

Figure S39. DFT-optimized geometries for cyclohexane dehydrogenation on Pt(111). Adsorption configuration diagram of cyclohexane dehydrogenation on Pt(111). Color code: Pt, blue; C, gray; H, white.

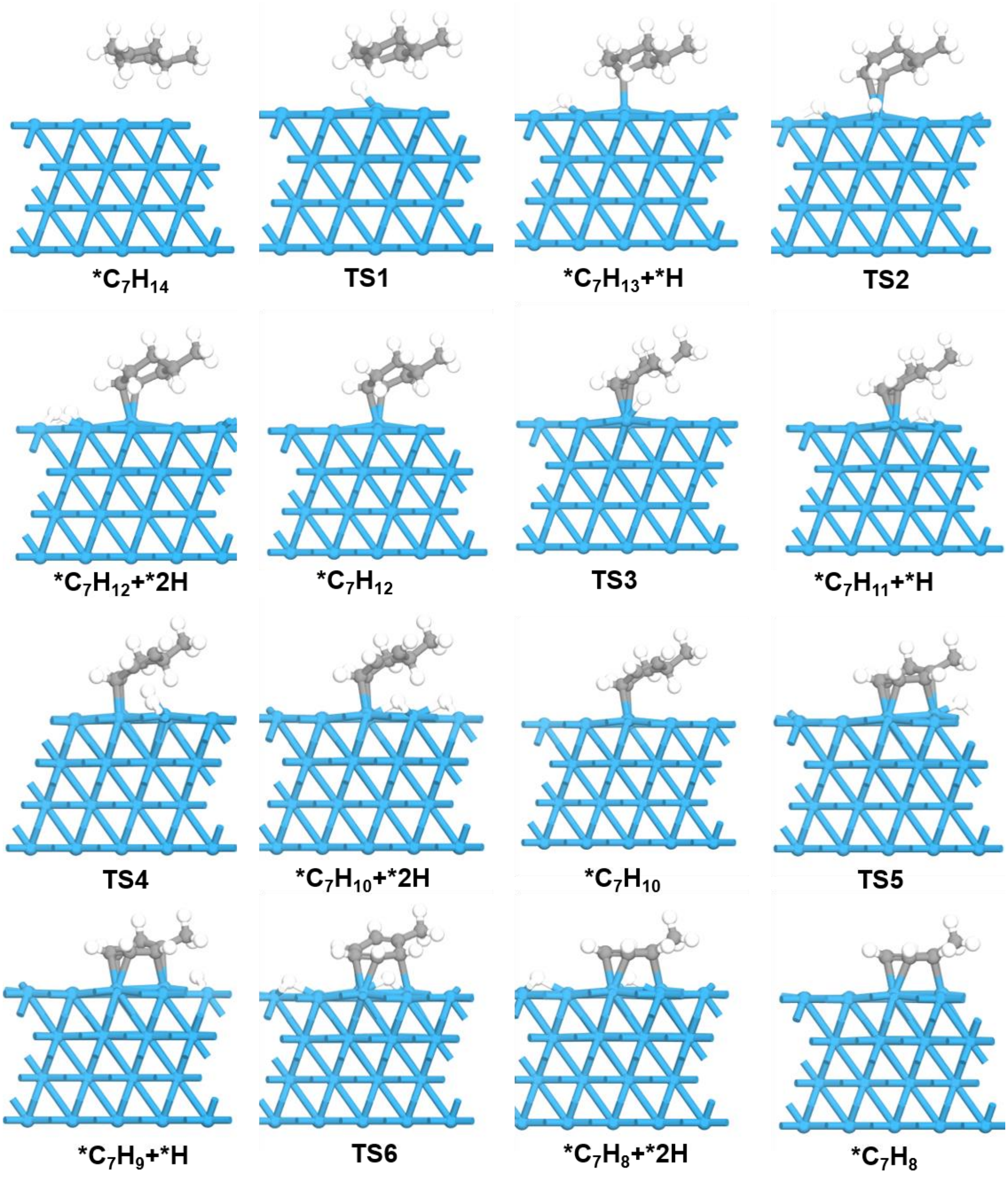

Figure S40. DFT-optimized geometries for methylcyclohexane dehydrogenation on Pt(111). Adsorption configuration diagram of methylcyclohexane dehydrogenation on Pt(111). Color code: Pt, blue; C, gray; H, white.

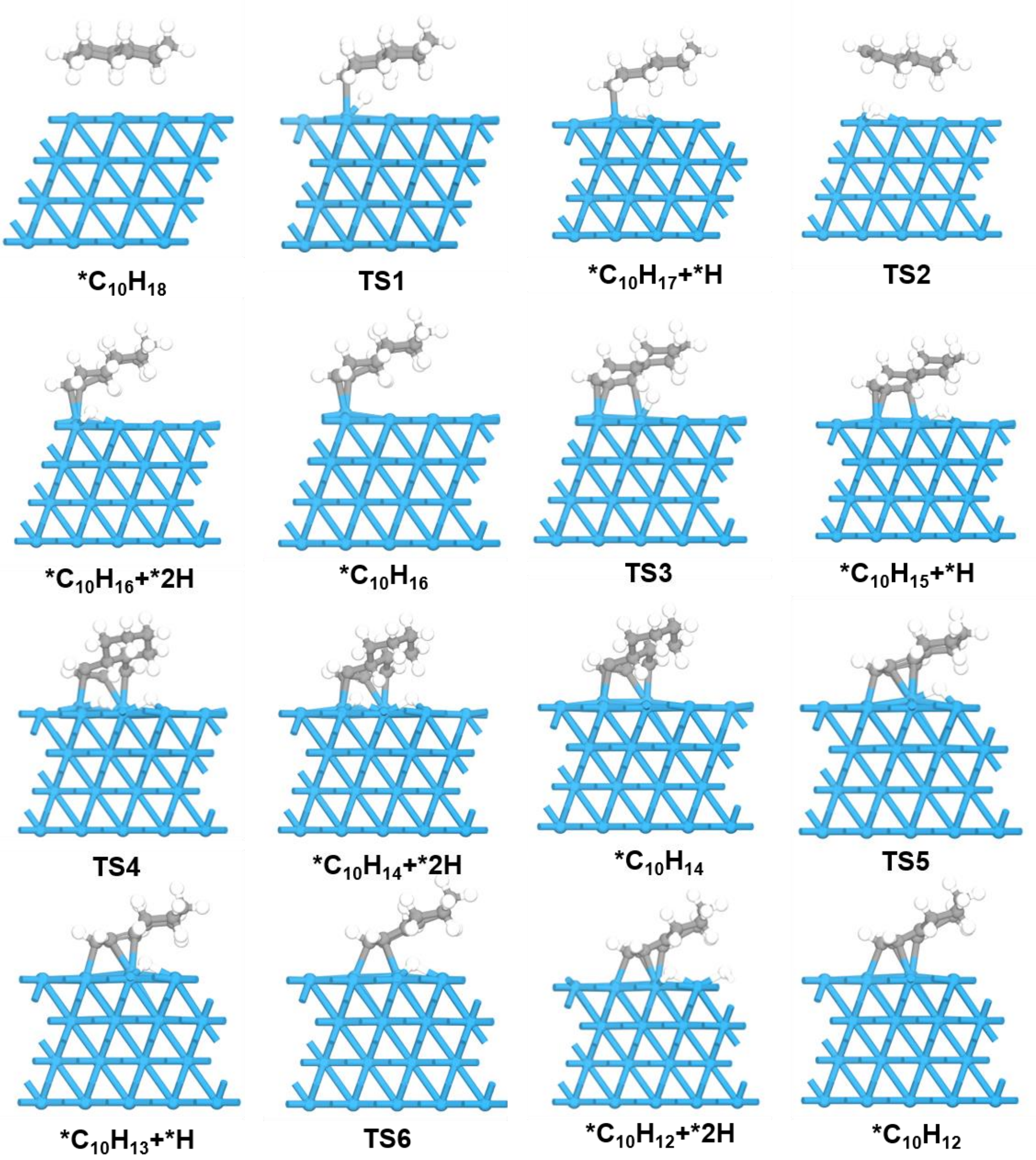

Figure S41. DFT-optimized geometries for decalin dehydrogenation on Pt(111).

Adsorption configuration diagram of decalin dehydrogenation on Pt(111). Color code: Pt, blue; C, gray; H, white.

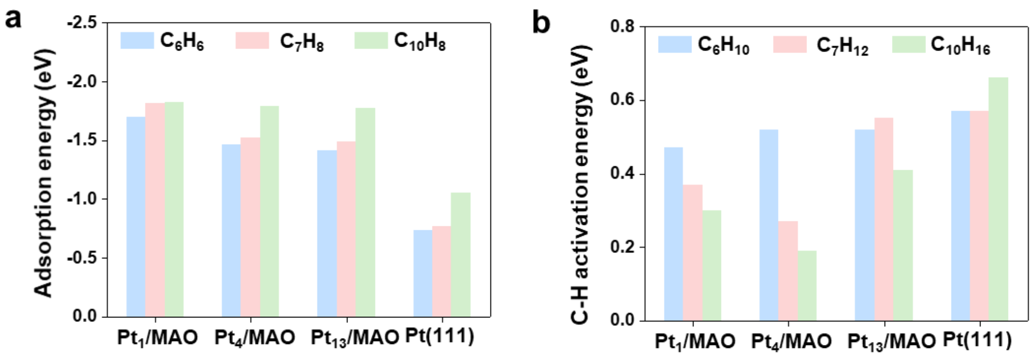

Figure S42. Energy trends for dehydrogenation on Pt catalysts. (a) Adsorption energy of aromatic products (C_6H_6, C_7H_8, and $C_{10}H_8$) and (b) C-H activation barriers of partially dehydrogenated intermediates (C_6H_{10}, C_7H_{12}, and $C_{10}H_{16}$) on various Pt catalyst models.

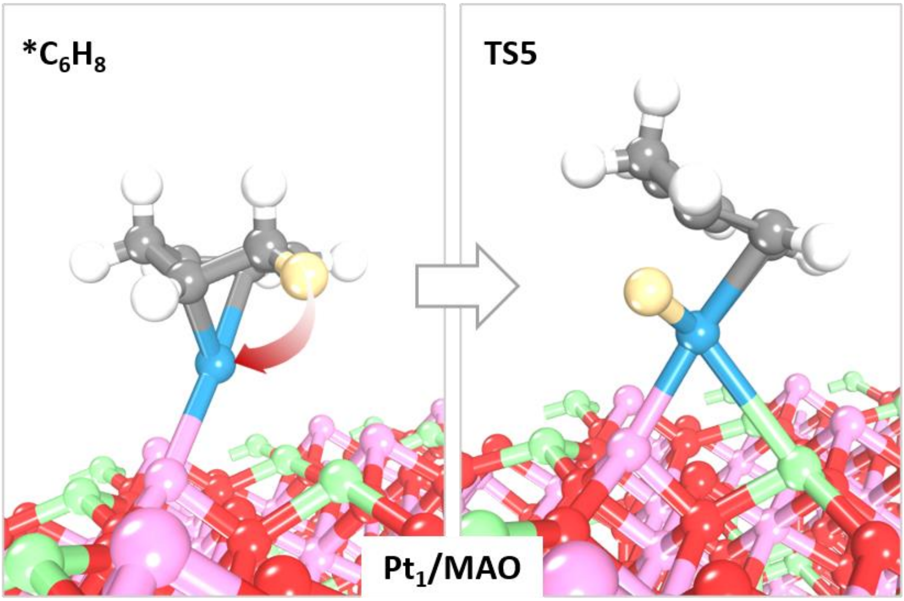

Figure S43. Rate-determining transition state for C-H activation. Structural representations of the rate-determining transition states on the Pt_1/MAO for the dehydrogenation of cyclohexane. Color code: Pt, blue; O, red; Al, pink; Mg, green; C, gray; H, white.

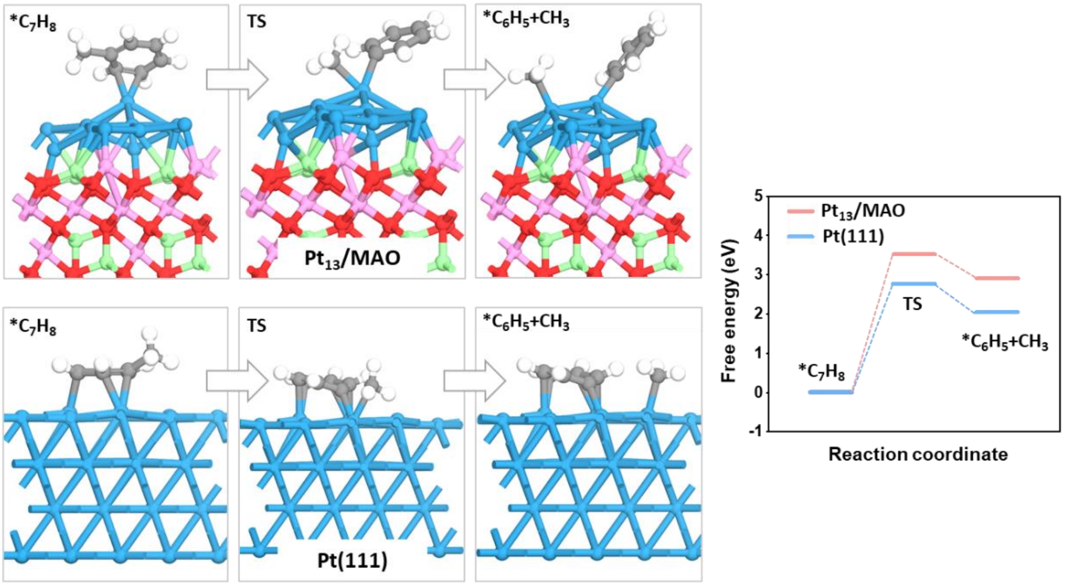

Figure S44. C_7H_8 demethylation on Pt_{13}/MAO and Pt(111). Reaction pathway and free energy profile of C_7H_8 demethylation on Pt_{13}/MAO and Pt(111). Color code: Pt, blue; O, red; Al, pink; Mg, green; C, gray; H, white.

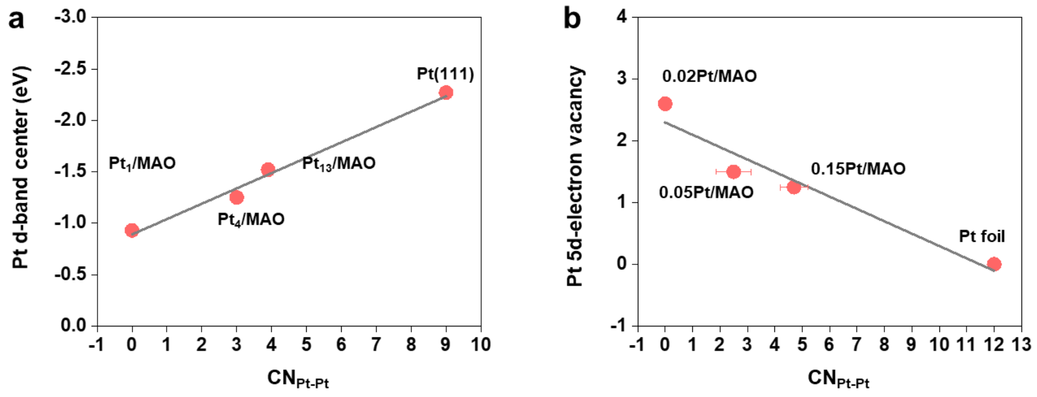

Figure S45. Correlation between coordination number, d-band center, and Pt 5d electron vacancy. Correlations of CN_{Pt-Pt} with (a) Pt d-band center from DFT calculations and (b) Pt 5d-electron vacancy from EXAFs analysis. Error bars represent 95% confidence intervals of the calculated CN_{Pt-Pt}.

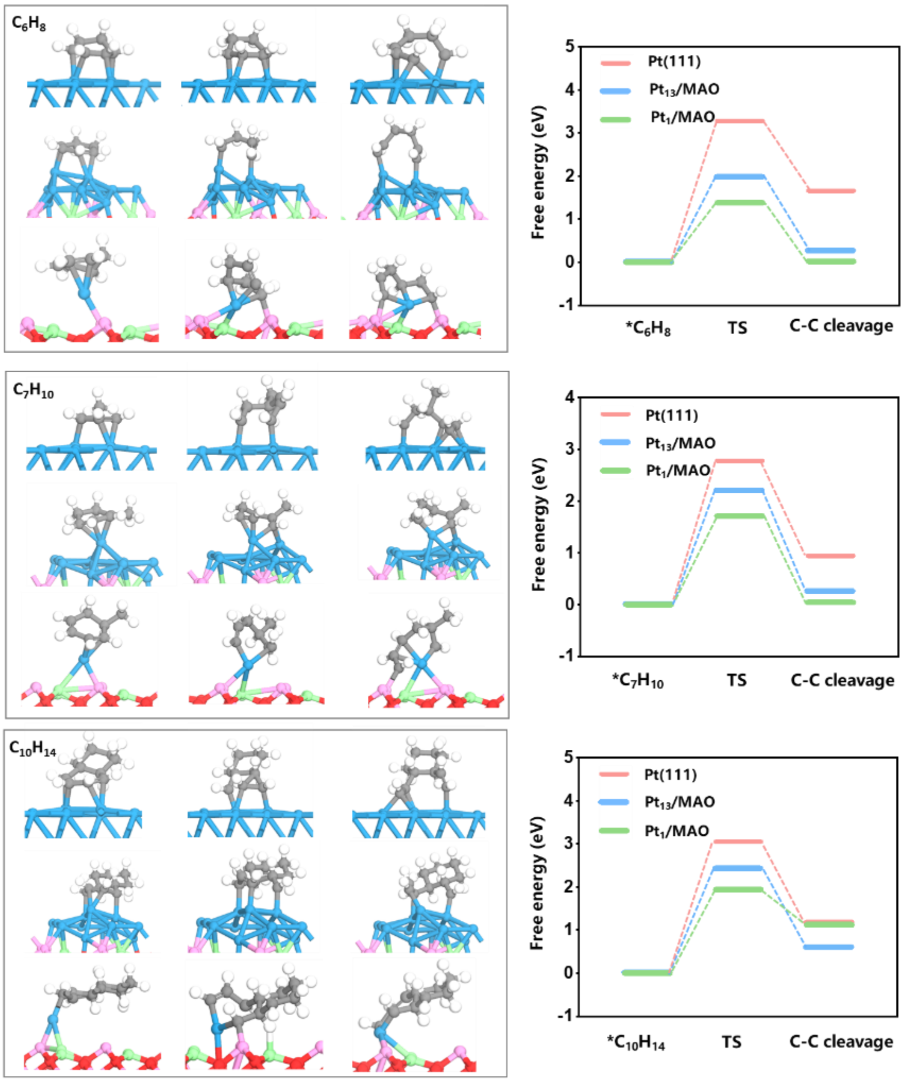

Figure S46. C-C bond cleavage on Pt catalysts. Reaction pathways and free energy profiles for C-C bond cleavage of C_6H_8, C_7H_{10}, $C_{10}H_{14}$ on Pt(111), Pt_{13}/MAO and Pt_1/MAO.

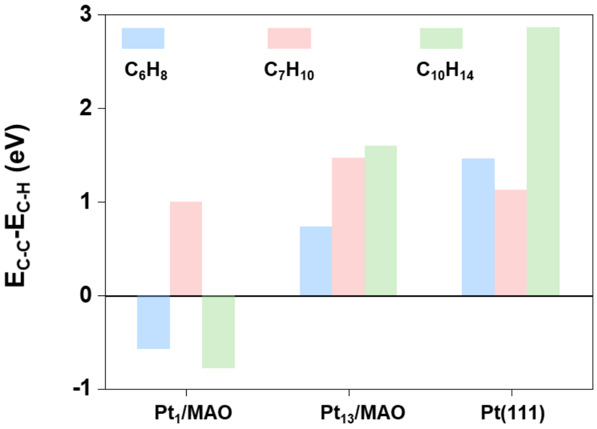

Figure S47. Selectivity trends on Pt catalysts. Selectivity descriptor ($\Delta E = E_{C\text{-}C} - E_{C\text{-}H}$) on Pt(111), Pt_{13}/MAO and Pt_1/MAO for partially dehydrogenated intermediates (C_6H_8, C_7H_{10}, $C_{10}H_{14}$).

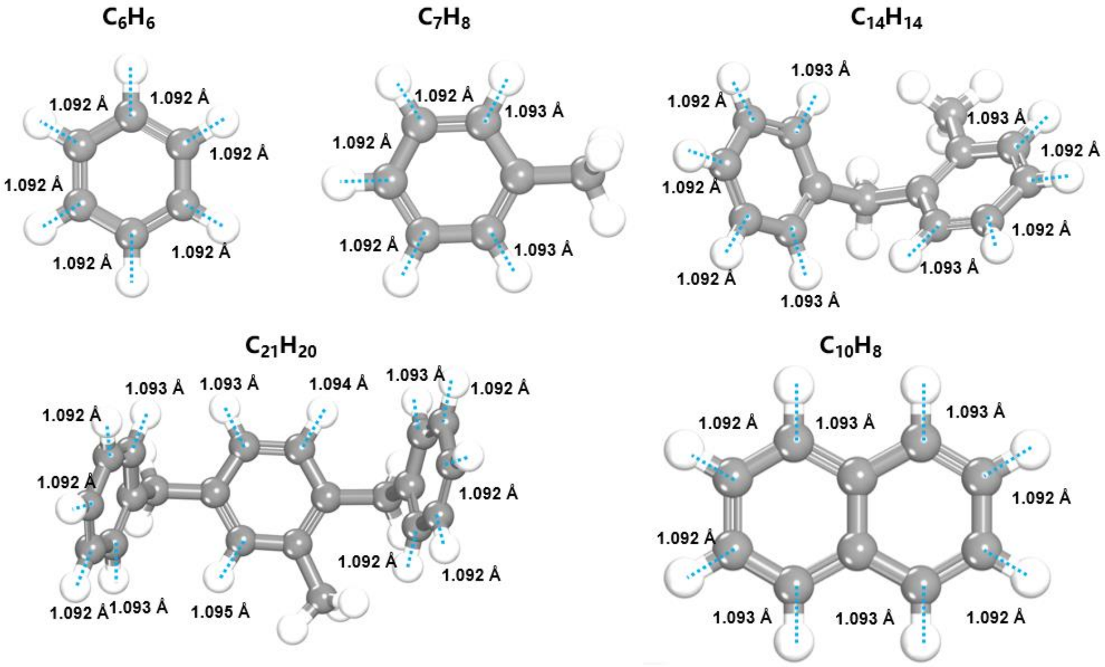

Figure S48. Molecular geometries of dehydrogenation products. The molecular structure of dehydrogenation products: benzene (C_6H_6), toluene (C_7H_8), benzyltoluene ($C_{14}H_{14}$), dibenzyltoluene ($C_{21}H_{20}$), and naphthalene ($C_{10}H_8$).

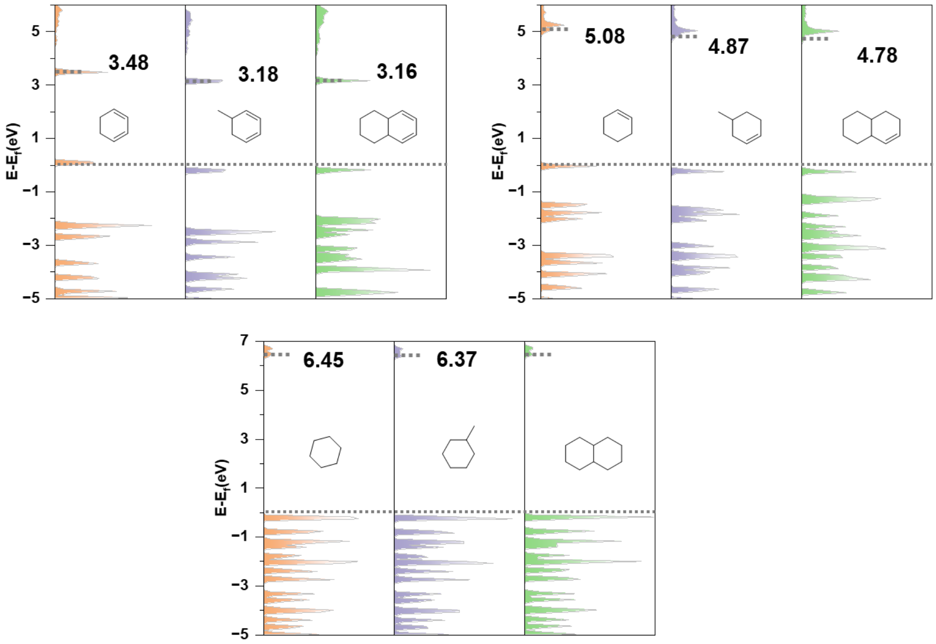

Figure S49. LUMO energy comparison. Projected density of states and LUMO energy of reactants and partially dehydrogenated intermediates.

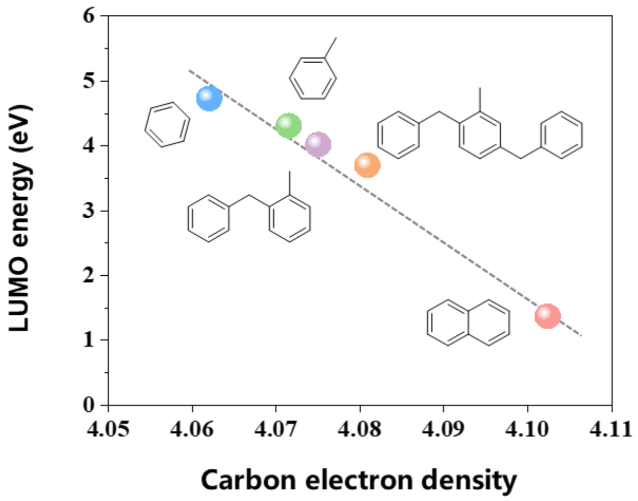

Figure S50. Electronic properties of dehydrogenation products. Correlation between carbon electron density and LUMO energy of aromatics.

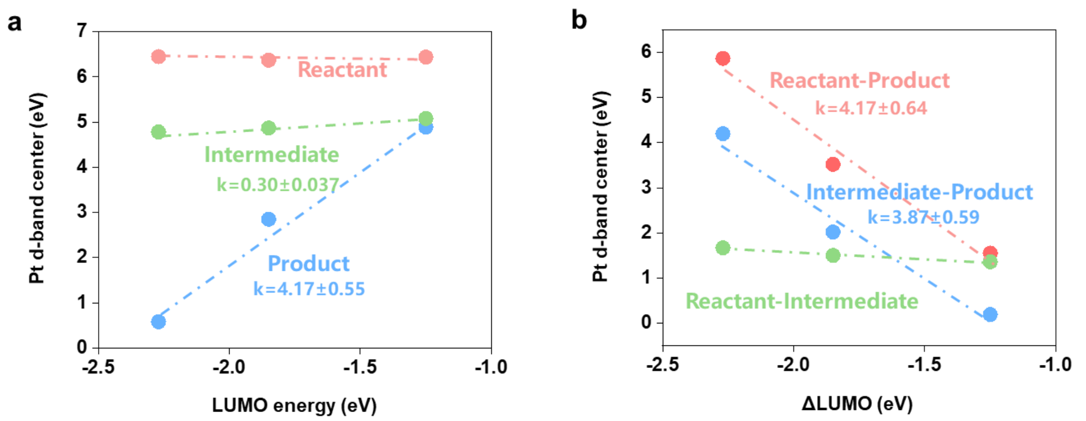

Figure S51. Relationship between Pt d-band center and LUMO energy along dehydrogenation pathways. (a) Correlation of the optimal Pt d-band center with the LUMO energy of reactants, partially dehydrogenated intermediates, and products. (b) Correlation of the optimal Pt d-band center with the ΔLUMO values along different reaction steps.

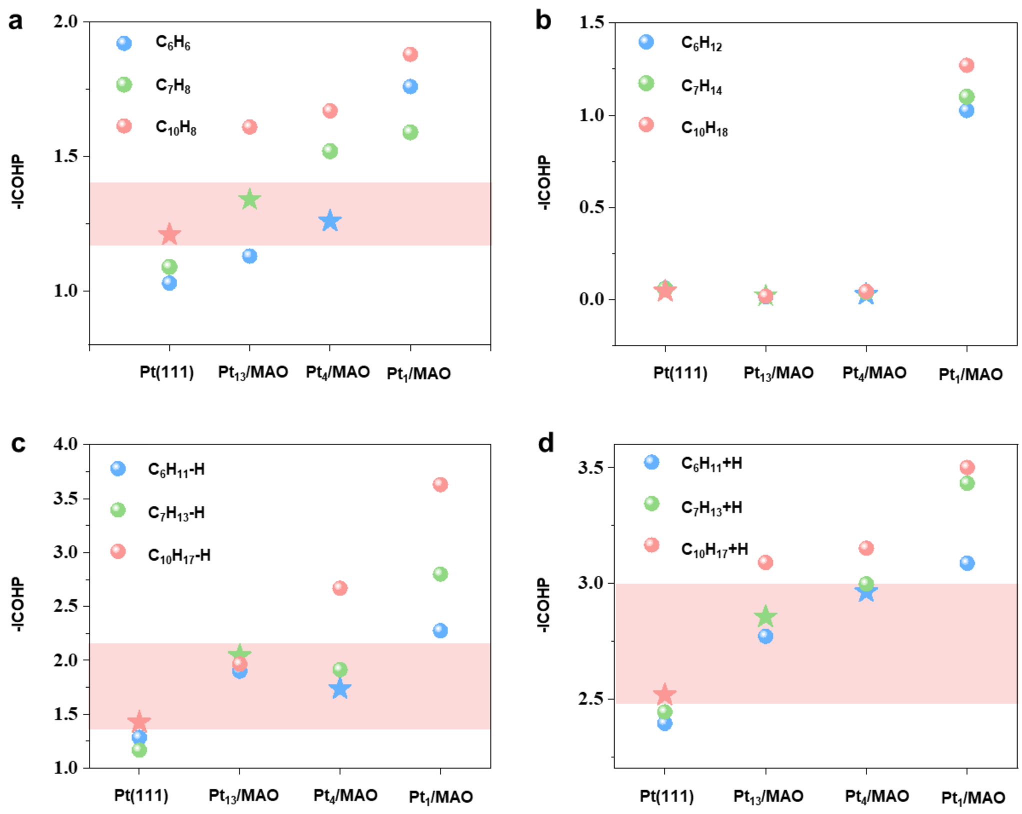

Figure S52. Bonding strength analysis of Pt-C interactions during key reaction steps. -ICOHP values of Pt-C bond for products adsorption (a), reactants adsorption (b), C-H activation (c), and intermediates adsorption (d) on various Pt catalyst models. The red-shaded regions indicate the optimal -ICOHP ranges.

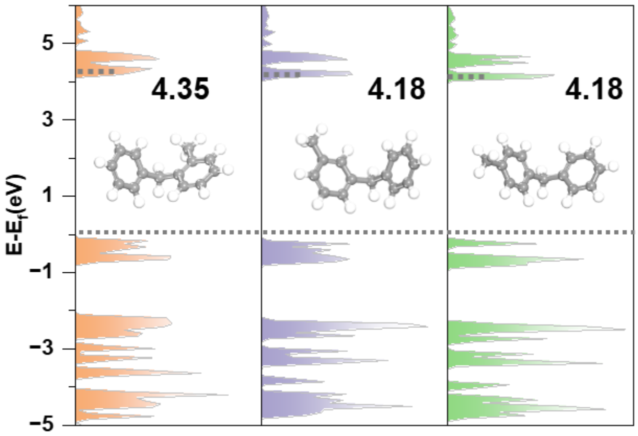

Figure S53. LUMO energy comparison of benzyltoluene isomers. Projected density of states and LUMO energy of 2-, 3-, 4-benzyltoluene isomers.

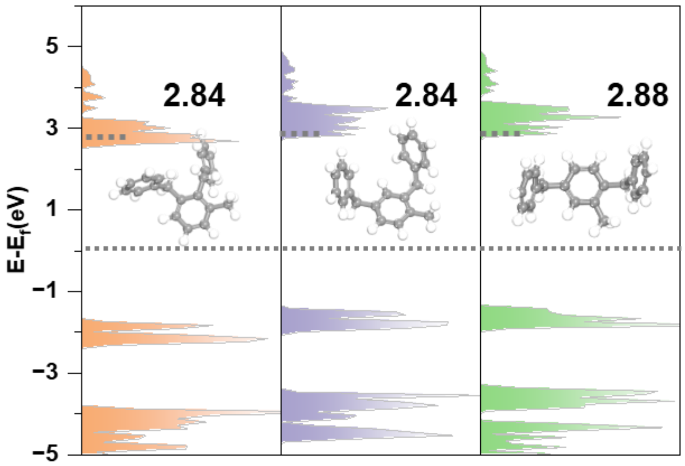

Figure S54. LUMO energy comparison of dibenzyltoluene isomers. Projected density of states and LUMO energy of 2,3-, 2,4-, 2,5-dibenzyltoluene isomers.

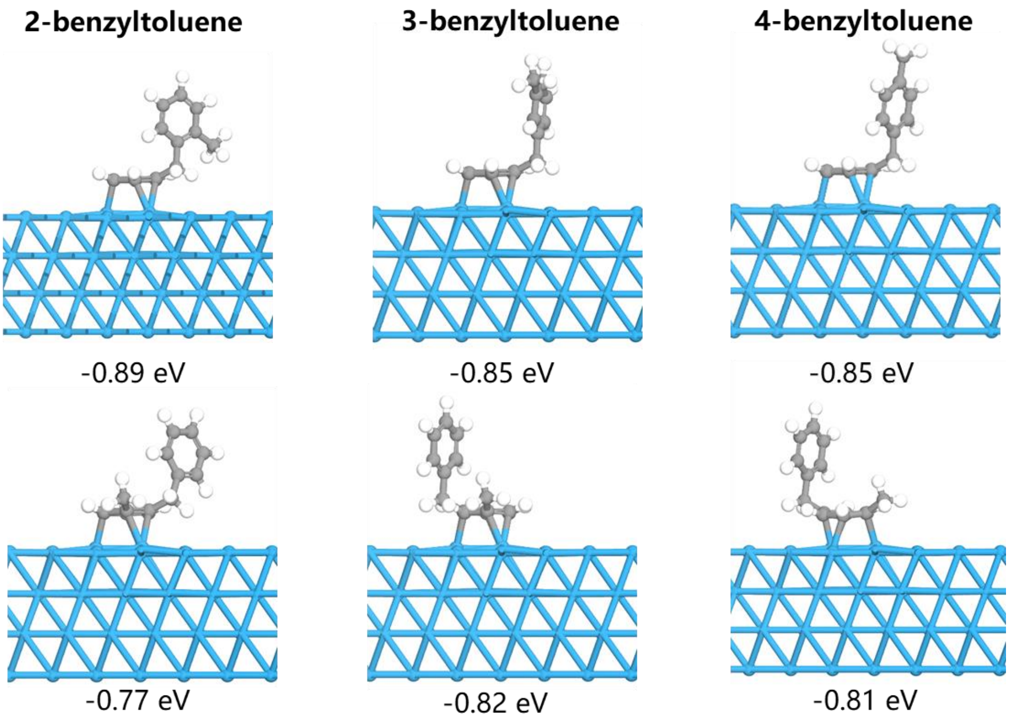

Figure S55. Adsorption behavior of benzyltoluene isomers on Pt surfaces.

Adsorption configurations and energies of benzyltoluene isomers on Pt(111).

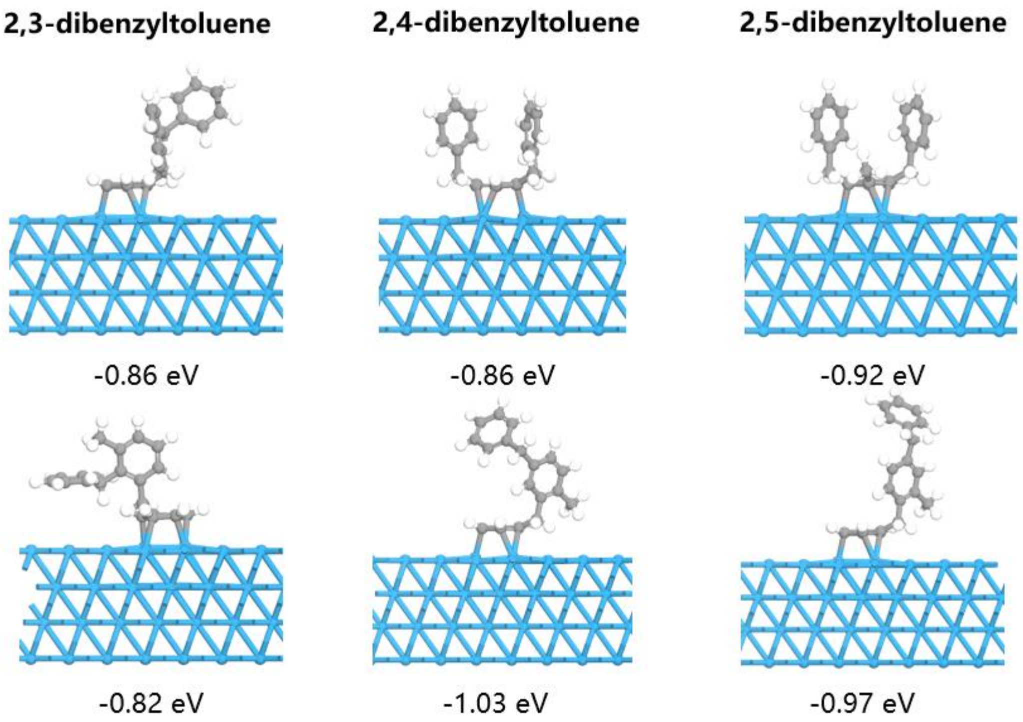

Figure S56. Adsorption behavior of dibenzyltoluene isomers on Pt surfaces.

Adsorption configurations and energies of dibenzyltoluene isomers on Pt(111).

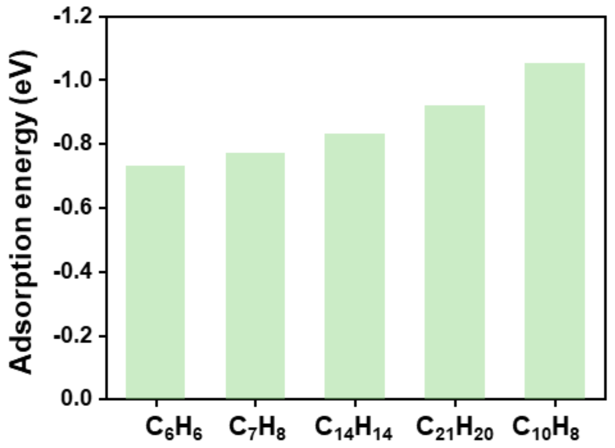

Figure S57. Adsorption energy trends of dehydrogenation products. Adsorption energy of aromatic products (C_6H_6, C_7H_8, $C_{14}H_{14}$, $C_{21}H_{20}$ and $C_{10}H_8$) on Pt(111) catalyst models.

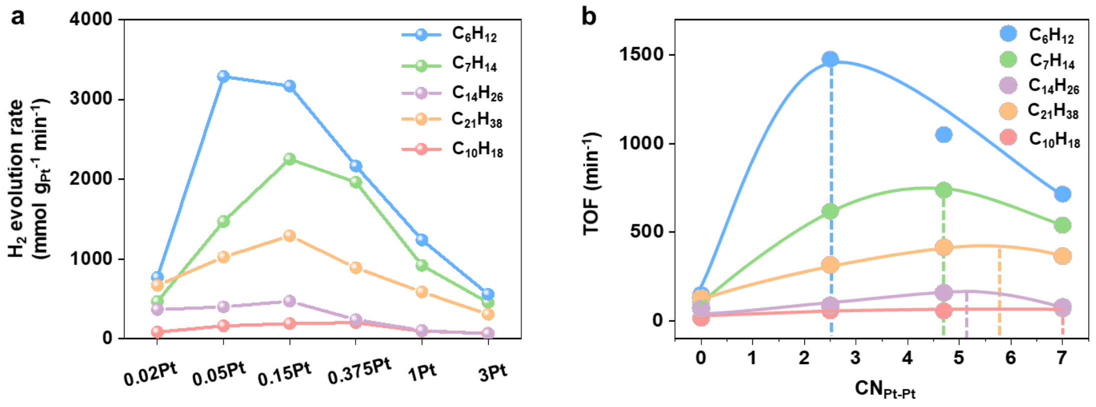

Figure S58. **Reactant structure-dependent dehydrogenation activity of Pt/MAO catalysts.** (a) Hydrogen evolution rates of Pt/MAO catalysts for the dehydrogenation of cyclohexane, methylcyclohexane, perhydro-benzyltoluene, perhydro-dibenzyltoluene and decalin at 280 °C. (b) Site-specific TOF as a function of $CN_{Pt\text{-}Pt}$ for different dehydrogenation reactions at 280 °C with polynomial fitting.

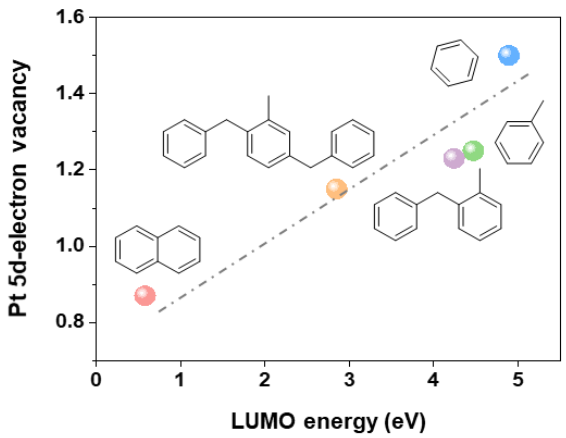

Figure S59. Electronic matching between Pt active sites and aromatic product orbitals. Correlation between the Pt 5d-electron vacancy of optimal catalyst and LUMO energy of aromatic product for different cycloalkane dehydrogenation reactions.

II. Supplementary Tables

Table S1. BET surface area, pore volume, and average pore diameter of Pt/MAO catalysts with varying Pt loadings.

Catalyst	S_{BET} ($cm^2 \cdot g^{-1}$)	V_p ($cm^3 \cdot g^{-1}$)	D_p (nm)
MAO	217.2	0.25	4.2
0.02Pt/MAO	156.3	0.34	6.6
0.05Pt/MAO	169.9	0.33	6.1
0.15Pt/MAO	170.8	0.33	6.1
0.375Pt/MAO	156.7	0.33	6.6
1Pt/MAO	170.1	0.32	5.8
3Pt/MAO	173.1	0.31	6.0

Table S2. Physic-chemical characteristics of the as-prepared Pt/MAO catalysts.

Catalyst	Pt loading (wt.%) [a]	Average Pt size (nm) [b]	Pt dispersion (%) [c]
0.02Pt/MAO	0.0228	-	100
0.05Pt/MAO	0.0514	-	69.06
0.15Pt/MAO	0.1438	-	48.45
0.375Pt/MAO	0.3872	1.40±0.39	36.72
1Pt/MAO	0.9990	1.70±0.49	11.94
3Pt/MAO	2.9904	2.45±0.81	4.14

[a] Obtained from ICP-AES measurement.

[b] Determined by HADDF-STEM characterization.

[c] Calculated by CO-chemisorption characterization.

Table S3. Comparative analysis of cyclohexane dehydrogenation performance across reported catalysts

Catalyst	Temperature (K)	Reactor	H_2 evolution rate (mmol g_{metal}^{-1} min^{-1})	Ref
Pt/CN	483	batch-wise reactor	34.0	[1]
Pt/ND@G	553	fixed bed reactor	1817.7	[2]
Ag-Rh/ACC	573	spray-pulse reactor	400.0	[3]
NiCu/SiO_2	623	fixed bed reactor	4.9	[4]
Pd/γ-Al_2O_3	673	fixed bed reactor	2923.0	[5]
Pt/MAO	553	fixed bed reactor	3284.0	This work
Pt/MAO	563	fixed bed reactor	5037.0	This work
Pt/MAO	573	fixed bed reactor	6848.0	This work

Table S4. Comparative analysis of methylcyclohexane dehydrogenation performance across reported catalysts.

Catalyst	Temperature (K)	Reactor	H_2 evolution rate (mmol g_{metal}^{-1} min^{-1})	Ref
Pt-Se/TiO_2	553	fixed bed reactor	143.0	[6]
Pt/SiO_2	573	fixed bed reactor	542.0	[7]
Pt/Al_2O_3	573	fixed bed reactor	656.1	[8]
Pt/GAC	573	fixed bed reactor	160.2	[9]
Pt-K/Al_2O_3	593	fixed bed reactor	242.7	[10]
Pt/Mg-Al-O	623	fixed bed reactor	1892.3	[11]
Pt-B/Al_2O_3	623	fixed bed reactor	961.0	[12]
Pt-Cu/S-1	623	fixed bed reactor	288.9	[13]
Pt-Mn/Al_2O_3	623	fixed bed reactor	369.0	[14]
Pt/CeO_2	623	fixed bed reactor	2509.0	[15]
Pt/MAO	553	fixed bed reactor	2251.0	This work
Pt/MAO	563	fixed bed reactor	3308.0	This work
Pt/MAO	573	fixed bed reactor	4276	This work

Table S5. Comparative analysis of decalin dehydrogenation performance across reported catalysts.

Catalyst	Temperature (K)	Reactor	H_2 evolution rate (mmol g_{metal}^{-1} min^{-1})	Ref
Pt/CNT	458	batch reactor	25.3	[16]
Pt/CNT-N	458	batch reactor	46.6	[16]
Pt/CNF	513	batch reactor	34.0	[17]
Pt/AC	533	batch reactor	10.2	[18]
Pt/Al_2O_3	533	batch reactor	20.8	[18]
Pt/C	533	batch reactor	52.9	[19]
Pt-Ni/C	563	batch reactor	95.9	[20]
Pt/MAO	553	fixed bed reactor	203.0	This work
Pt/MAO	563	fixed bed reactor	321.0	This work
Pt/MAO	573	fixed bed reactor	599.0	This work

Table S6. Least-squares fitting details for determining the fractions of Pt species in 0.05Pt/MAO and 0.15Pt/MAO catalysts based on kinetic data.

**Catalyst**	**f_s**	**f_c**	**f_n**	**x**	**CYH**		**MCH**		**DEC**		**R^2**
					**E_a**	**lnA**	**E_a**	**lnA**	**E_a**	**lnA**	
0.05Pt/MAO	0.63	0.37	1.7×10^{-15}	0.85	138.57	38.47	103.47	31.55	102.11	29.41	0.9963
0.15Pt/MAO	1.68×10^{-15}	0.56	0.44	0.73	74.17	24.54	129.63	36.08	200.00	49.42	0.9973

Table S7. EXAFS fitting parameters at the Pt L_{III}-edge for various Pt samples (S_0^2=0.90).

Sample	Shell[a]	CN[b]	R(Å)[c]	σ^2 (Å^2)[d]	ΔE_0 (eV)[e]	*R* factor
**Pt foil**	Pt-Pt	12	2.76±0.01	0.003±0.001	8.48±0.31	0.001
**0.02Pt/MAO**	Pt-O	4.07±1.0	1.93±0.04	0.009±0.007	9.26±3.43	0.036
**0.05Pt/MAO**	Pt-O	3.75±0.8	1.86±0.03	0.004±0.001	7.14±3.9	0.009
	Pt-Pt	0.93±0.2	2.58±0.03	0.004±0.001		
**0.15Pt/MAO**	Pt-O	1.90±0.26	1.97±0.02	0.001±0.001	7.88±2.25	0.041
	Pt-Pt	5.30±0.58	2.71±0.05	0.003±0.001		

[a] The EXAFS fitting employed single-scattering paths for both the Pt-O and Pt-Pt contributions, derived from PtO_2 and Pt foil reference structures, respectively.

[b]*CN*: coordination number; [c]*R*: distance between absorber and backscatter atoms; [d]σ^2: Debye-Waller factor to account for both thermal and structural disorders; [e]ΔE_0: inner potential correction.

R factor indicates the goodness of the fit. Fitting range: 3 < k (Å^{-1}) < 11 and 1.0 < R (Å)< 3.6. No inter-shell constraints or tie-lines were applied.

III. Supplementary References

1. Wang, J. et al. Size-Dependent Catalytic Cyclohexane Dehydrogenation with Platinum Nanoparticles on Nitrogen-Doped Carbon. *Energ. Fuel.* **34**, 16542-16551 (2020).
2. Deng, Y. et al. Few-atom Pt ensembles enable efficient catalytic cyclohexane dehydrogenation for hydrogen production. *J. Am. Chem. Soc.* **144**, 3535-3542 (2022).
3. Pande, J.V., Bindwal, A.B., Pakade, Y.B. & Biniwale, R.B. Application of microwave synthesized Ag-Rh nanoparticles in cyclohexane dehydrogenation for enhanced H_2 delivery. *Int. J. Hydrogen Energy* **43**, 7411-7423 (2018).
4. Xia, Z., Lu, H., Liu, H., Zhang, Z. & Chen, Y. Cyclohexane dehydrogenation over Ni-Cu/SiO2 catalyst: Effect of copper addition. *Catal. Commun.* **90**, 39-42 (2017).
5. Zhou, G. et al. Reversible hydrogenation and dehydrogenation of benzene for hydrogen storage on highly dispersed Pd/γ-Al2O3 catalyst. *J. Ind. Eng. Chem.* **134**, 561-573 (2024).
6. Ito, H. et al. Improved catalytic stability of Pt/TiO2 catalysts for methylcyclohexane dehydrogenation via selenium addition. *Int. J. Hydrogen Energy* **47**, 38635-38643 (2022).
7. Nakaya, Y., Miyazaki, M., Yamazoe, S., Shimizu, K.-i. & Furukawa, S. Active, Selective, and Durable Catalyst for Alkane Dehydrogenation Based on a Well-Designed Trimetallic Alloy. *ACS Catal.* **10**, 5163-5172 (2020).
8. Chen, C.-X. et al. Effect of Pt particle size on methylcyclohexane dehydrogenation over Pt/Al2O3 catalysts. *Fuel* **360**, 130607 (2024).
9. Ye, H.-L., Liu, S.-X., Zhang, C., Cai, Y.-Q. & Shi, Y.-F. Dehydrogenation of methylcyclohexane over Pt-based catalysts supported on functional granular activated carbon. *RSC Adv.* **11**, 29287-29297 (2021).
10. Han, S. et al. Synergistic structural and electronic influences of Pt bead catalysts

on dehydrogenation activity for liquid organic hydrogen carriers. *Chem. Eng. J.* **487** (2024).

11. Wu, K. et al. Preparation of Pt supported on mesoporous Mg–Al oxide catalysts for efficient dehydrogenation of methylcyclohexane. *Int. J. Hydrogen Energy* **46**, 25513-25519 (2021).
12. Wu, X. et al. Acid site introduced by Al3+penta and boron in Pt/Al2O3 catalyst for dehydrogenation of methylcyclohexane. *Int. J. Hydrogen Energy* **47**, 34955-34962 (2022).
13. Zhang, X. et al. Study of the carbon cycle of a hydrogen supply system over a supported Pt catalyst: methylcyclohexane–toluene–hydrogen cycle. *Catal. Sci. Technol.* **10**, 1171-1181 (2020).
14. Nakano, A. et al. Effects of Mn addition on dehydrogenation of methylcyclohexane over Pt/Al2O3 catalyst. *Appl. Catal., A* **543**, 75-81 (2017).
15. Chen, L. et al. Reversible dehydrogenation and rehydrogenation of cyclohexane and methylcyclohexane by single-site platinum catalyst. *Nat. Commun.* **13**, 1092 (2022).
16. Tuo, Y. et al. Carbon nanotubes-supported Pt catalysts for decalin dehydrogenation to release hydrogen: A comparison between nitrogen- and oxygen-surface modification. *Int. J. Hydrogen Energy* **46**, 930-942 (2021).
17. Lázaro, M.P., García-Bordejé, E., Sebastián, D., Lázaro, M.J. & Moliner, R. In situ hydrogen generation from cycloalkanes using a Pt/CNF catalyst. *Catal. Today* **138**, 203-209 (2008).
18. Wang, F. et al. Preparation of Pt/MgAl2O4 Decalin Dehydrogenation Catalyst for Chemical Hydrogen Storage Application. *Catal. Lett.* **154**, 191-205 (2023).
19. Sebastián, D., Bordejé, E.G., Calvillo, L., Lázaro, M.J. & Moliner, R. Hydrogen storage by decalin dehydrogenation/naphthalene hydrogenation pair over platinum catalysts supported on activated carbon. *Int. J. Hydrogen Energy* **33**, 1329-1334 (2008).

20. Qi, S. et al. Hydrogen production from decalin dehydrogenation over Pt-Ni/C bimetallic catalysts. *Chinese J. Catal.* **35**, 1833-1839 (2014).
